# Polycystic Ovary Syndrome and Obesity: A Cross-Sectional Survey of Patients and Obstetricians/Gynecologists

**DOI:** 10.1089/jwh.2022.0471

**Published:** 2023-06-06

**Authors:** Lisa Gill, Jamie E. Coborn, Anthony R. Hoovler, Katherine Sherif

**Affiliations:** ^1^ Department of Obstetrics, Gynecology and Women's Health, University of Minnesota, Minneapolis, Minnesota, USA.; ^2^ Novo Nordisk, Inc., Plainsboro, New Jersey, USA.; ^3^ Jefferson Women's Primary, Jefferson University, Philadelphia, Pennsylvania, USA.

**Keywords:** anti-obesity agents, patient care team, obesity management, self report, weight loss

## Abstract

**Background:**

Polycystic ovary syndrome (PCOS) is an endocrine disorder commonly affecting women of reproductive age. Compared with women without PCOS, women with PCOS are more likely to have overweight or obesity.

**Materials and Methods:**

To better understand the role of obstetricians/gynecologists (OB/GYNs) in diagnosis and treatment of patients with PCOS and obesity, we conducted an anonymous, United States population-based, cross-sectional online survey with 251 patients with PCOS and obesity and 305 health care professionals (HCPs), of which 125 were OB/GYNs.

**Results:**

In the most common patient journey, most patients were diagnosed (66%) and treated (59%) by OB/GYNs. Most patients (51%) considered OB/GYNs to be the coordinator of their PCOS care. For ongoing management of patients with PCOS and obesity, OB/GYNs reported prescribing general improvements in lifestyle (91%), oral contraceptives (91%), metformin (85%), letrozole (74%), spironolactone (71%), specific diets (60%), medroxyprogesterone (45%), and anti-obesity medications (27%). OB/GYNs were significantly more likely than other HCPs surveyed to strongly agree with the statement that they do not know enough about anti-obesity medications to feel comfortable prescribing them to their patients with PCOS and obesity (*p* < 0.05). Most OB/GYNs believed that consultation with a dietitian/nutritionist (75%) or access to a physician who specializes in obesity (67%) were the most beneficial types of support for their patients with PCOS and obesity.

**Conclusions:**

OB/GYNs recognize the importance of obesity management for the treatment of PCOS; however, utilization of effective obesity tools to treat these patients is low. OB/GYNs may benefit from additional education on obesity management strategies.

## Introduction

Polycystic ovary syndrome (PCOS) affects 5%–20% of reproductive aged women in the world.^1^ Women with PCOS may present with hyperandrogenism, hirsutism, ovulatory dysfunction with abnormal menses, and/or polycystic ovarian morphology.^1^ PCOS is frequently associated with obesity and other metabolic disorders. The prevalence of obesity in women with PCOS is estimated at 49% (95% confidence interval: 42%–55%).^2^

PCOS is suspected to contribute to obesity through insulin resistance and androgen excess.^3^ Many women with PCOS have insulin resistance, which contributes to metabolic dysfunction^4^ and compensatory hyperinsulinemia.^5^ Hyperinsulinemia causes disruptions in the hypothalamic-pituitary-ovarian axis, which leads to overproduction of luteinizing hormone,^6^ resulting in increased androgen production in the theca cells of the ovaries.^7^ Hyperinsulinemia also stimulates corticotropin-mediated adrenal androgen production and inhibits synthesis of sex-hormone-binding globulin, resulting in increased free androgen levels.^7^

Androgen excess is suspected to initiate a positive feedback loop of abdominal fat accumulation, which, in turn, triggers further increased androgen production and insulin resistance.^3,8^ Thereby, insulin resistance and androgen excess create a positive feedback loop of metabolic dysregulation that predisposes women with PCOS to obesity.

The American College of Obstetricians and Gynecologists (ACOG) recommends that androgen excess is managed with hormonal contraceptives if women are not seeking pregnancy. However, lifestyle change focused on diet and exercise is recommended as a long-term solution for women with PCOS and obesity, as weight loss ameliorates many symptoms of PCOS.^9^ For example, weight loss through bariatric surgery has been shown to resolve PCOS in many patients.^10^

Lifestyle intervention coupled with metformin has also shown efficacy for PCOS management; lifestyle intervention and metformin were associated with lower body mass index (BMI), less subcutaneous adipose tissue, and increased number of menstrual cycles after 6 months when compared with lifestyle plus placebo.^11^ PCOS has been shown to be primarily diagnosed by obstetricians/gynecologists (OB/GYNs).^12^ Previous survey research has revealed that patients are dissatisfied with the PCOS diagnostic process^12,13^ and knowledge gaps exist in OB/GYNs regarding diagnosis and management of patients with PCOS.^14^

Therefore, our study aimed at understanding the typical medical journey of patients with PCOS and obesity, the role of OB/GYNs in diagnosing and treating patients with PCOS and obesity, and at identifying potential gaps in treatment of patients with PCOS and obesity by OB/GYNs, specifically with regards to obesity management.

## Materials and Methods

### Study design and participants

A cross-sectional study consisting of an anonymous, U.S.-population based, national online survey was conducted among patients with obesity diagnosed with PCOS and health care professionals (HCPs) treating patients with PCOS and obesity.

Data were collected from October 30, 2020, to December 1, 2020. All respondents were recruited via email by an online panel company to which respondents had provided permission to be contacted for research purposes. Respondents were informed of the nature of the research, that their participation in the research was voluntary, and that they could withdraw at any time before consenting to the survey terms.

Respondents answered screening questions to determine eligibility for the study. Recruitment continued until predetermined targets for each respondent type were met. We intentionally surveyed more OB/GYNs than other HCPs because they are considered the primary care coordinators for patients with PCOS, and we hypothesized that they received the most referrals for diagnosis and treatment of patients with PCOS.

Eligible participants completing the entire survey received a modest monetary incentive. The study protocol was submitted to the Western Institutional Review Board for ethical approval and was determined to be exempt, because the research includes survey procedures with adequate provisions to protect the privacy of participants and maintain data confidentiality. Responses were kept strictly confidential and never associated with a respondent's name. This research adhered to ethical standards for human experimentation established in the Declaration of Helsinki.

Unique surveys were developed based on extensive literature review and qualitative interviews with HCPs and patients with PCOS and obesity. The qualitative interviews consisted of 60-minute in-depth telephone interviews with HCPs and patients with PCOS. Similar discussion guides were used for both the HCPs and patients to determine gaps and areas of alignment relating to PCOS care. The survey was developed and conducted by a third-party vendor (KJT Group, Inc., Rochester, NY) with oversight from the authors. Gaps identified in the literature and areas requiring further exploration and quantification were identified and included in the surveys.

Separate surveys were used for patients (Supplementary Appendix Survey SA1) and HCPs (Supplementary Appendix Survey SA2) to measure attitudes and experiences with PCOS before diagnosis; experience with the diagnostic process; management and treatment of PCOS; PCOS management guidelines (HCPs); obesity discussions, management, and attitudes; and informational sources for PCOS and obesity.

The surveys consisted of a variety of yes/no, multiple-choice, and Likert-scale questions (on a scale from one to seven). The study samples were independent, that is, patients and HCPs surveyed were not matched pairs. Patients were included if they were U.S. residents, aged 18–55 years, had a self-reported diagnosis of PCOS, were born female and identified as female, were seeking help with fertility or other PCOS-related issues (excess weight, hirsutism, irregular menses), and had obesity (BMI ≥30 kg/m^2^).

The HCPs included physicians employed in U.S. facilities (except Maine and Vermont to comply with Sunshine Act reporting requirements), physicians practicing as a primary care physician (internal medicine, family practice, and general practice), OB/GYNs, general endocrinologists, or reproductive endocrinologists. The HCPs treated at least five patients (primary care physicians and general endocrinologists) or ten patients (OB/GYNs and reproductive endocrinologists) in the past month with PCOS and obesity. The HCPs had board certification or eligibility in their chosen specialty, were in practice for 3–25 years, and not practicing in a government facility or an ambulatory surgical center.

### Statistical analyses

We performed descriptive statistical analysis (means, frequencies) using SPSS Statistics for Windows 23 (SPSS, Chicago, IL). Tests of differences (chi square, *t*-tests) within respondent types were performed using SPSS. Statistical significance was set at *p* < 0.05, using two-tailed tests. Data are presented as number and percentage for categorical variables, and continuous data expressed as the mean ± standard deviation (SD) unless otherwise specified.

For certain survey questions, we wanted to understand the medical journey that patients had taken to receive a diagnosis of PCOS. We asked patients to list the order in which they had discussions about possible PCOS symptoms, received treatment for their PCOS, and received an official PCOS diagnosis. The most common patient journey included patients who had discussions about symptoms, followed by a PCOS diagnosis, and treatment. We refer to this subset of patients for several questions about the types of HCPs seen at each stage in the patient's medical journey.

## Results

Sample characteristics of HCPs and patients with PCOS and obesity are shown in Table 1. Most patients (88%) said they recalled the specific HCP who first told them that their symptoms may be PCOS. Among these patients, most (62%) said an OB/GYN was the physician to first mention potential PCOS. One third (33%) of patients reported experiencing misdiagnosis with another condition before their PCOS diagnosis, and 46% of these patients reported receiving a misdiagnosis from an OB/GYN. The misdiagnoses were identified most often as endometriosis or irregular/heavy periods.

**Table 1.** Sample Characteristics

Characteristics of survey respondents	Patients with PCOS and obesity (*N* = 251)	
Mean age (SD), years	34.8 (9.6)	
Age of symptom onset, years, *n* (%)		
<17	112 (45)	
18–30	118 (47)	
>30	21 (8)	
Ethnicity, *n* (%)^a^		
White	213 (85)	
Black/African American	22 (9)	
Spanish/Hispanic/Latino	20 (8)	
Other	20 (8)	
Education level, *n* (%)^b^		
High school diploma or equivalent (GED)	105 (42)	
Associate degree	55 (22)	
Bachelor's degree	68 (27)	
Master's degree or higher	20 (8)	
None of the above	3 (1)	
Prevalence of self-reported comorbidities, *n* (%)^a^		
Obesity	185 (74)	
Anxiety	170 (68)	
Depression	169 (67)	
Hypertension	70 (28)	
Type 2 diabetes	47 (19)	
Dyslipidemia	38 (15)	
BMI class, *n* (%)		
Class I (30 to <35 kg/m^2^)	76 (30)	
Class II (35 to <40 kg/m^2^)	59 (24)	
Class III (≥40 kg/m^2^)	116 (46)	

	Health care professionals (*n* = 305)	OB/GYNs (*n* = 125)
Setting, *n* (%)		
Urban	108 (35)	40 (32)
Suburban	169 (55)	75 (60)
Rural	28 (9)	10 (8)
Sex, *n* (%)		
Male	175 (57)	55 (44)
Female	129 (42)	69 (55)
Other	1 (<1)	1 (<1)
Mean time in practice, years	16.0	16.1
Professional specialty, *n* (%)		
OB/GYN	125 (41)	
General endocrinologist	75 (25)	
Reproductive endocrinologist	30 (10)	
Primary care^c^	75 (25)	

^a^ Percentages sum to greater than 100% as patients could select more than one option.

^b^ Percentages may not sum to 100% due to rounding.

^c^ Primary care physicians included physicians specializing in internal medicine, family practice, and general practice.

BMI, body mass index; OB/GYNs, obstetricians/gynecologists; PCOS, polycystic ovary syndrome; SD, standard deviation.

**FIG. 1.**
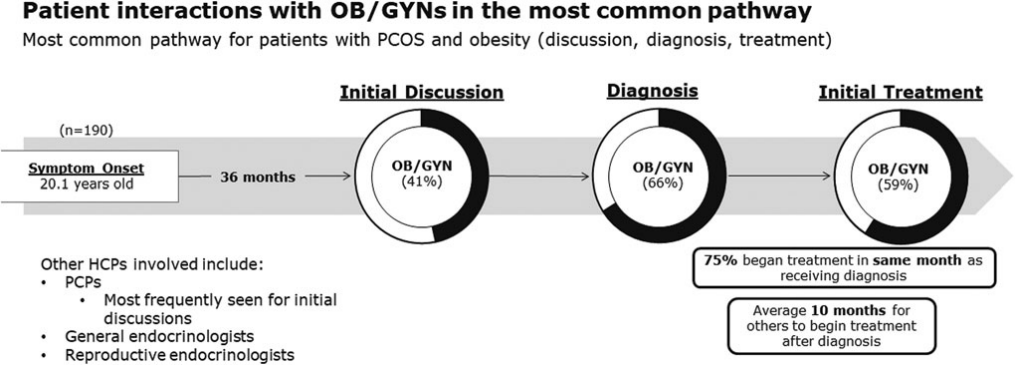
Patient interactions with OB/GYNs in the most common pathway. The most common pathway (comprising 76% of patients) relates to patients who had initial discussions with an HCP about their PCOS symptoms, followed by a PCOS diagnosis, and PCOS treatment. HCP, health care professional; OB/GYN; obstetrician/gynecologist; PCOS, polycystic ovary syndrome; PCP, primary care physician.

**FIG. 2.**
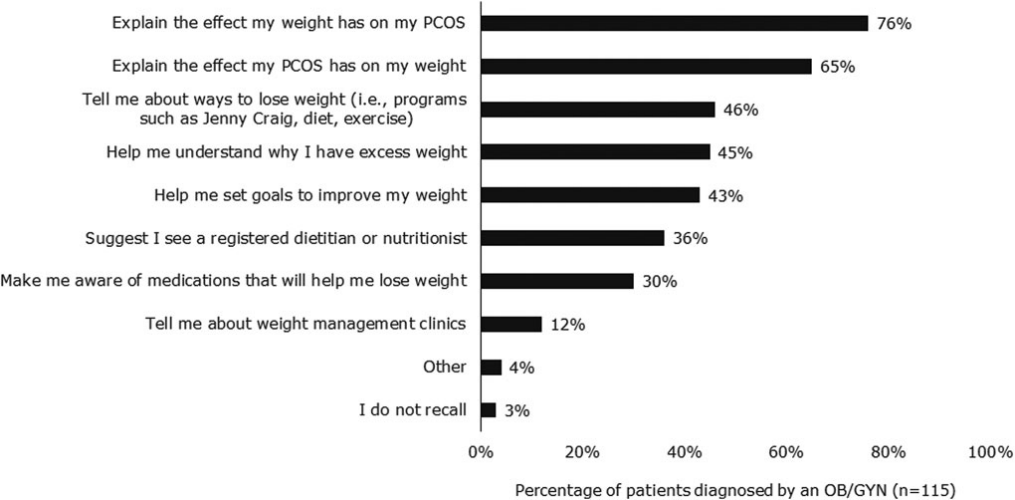
Weight management topics discussed at time of diagnosis. Among patients who said their providers discussed weight management at diagnosis, patients reported that OB/GYNs commonly discussed the effect weight has on PCOS and the effect of PCOS on weight. Few patients recalled discussions about weight loss clinics, anti-obesity medications, or seeing a dietitian/nutritionist.

In the most common patient journey (comprising 76% of patients), most patients were diagnosed (66%) and treated (59%) by OB/GYNs (Fig. 1). Among patients with PCOS and obesity diagnosed with PCOS by OB/GYNs, most said that their OB/GYN explained the impact that their excess weight has on their PCOS (76%) and explained the effect their PCOS has on their weight (65%) at the time of diagnosis (Fig. 2). Patients also reported that OB/GYNs discussed ways to lose weight (46%), why patients had excess weight (45%), goal setting to improve weight outcomes (43%), options to see a registered dietitian or nutritionist (36%), utility of anti-obesity medications (30%), and potential to visit a weight loss clinic (12%).

Most patients (75%) began treatment in the same month they were diagnosed. Half of the patients (51%) considered OB/GYNs to be the coordinator of their PCOS care with primary care professionals being the second most likely to be considered the PCOS care coordinator (19%). Almost one in five patients (18%) did not consider any HCP to be the coordinator of their PCOS care. Almost all OB/GYNs (94%) said they followed ACOG guidelines for the treatment of PCOS.

For ongoing management of patients with PCOS and obesity, OB/GYNs recommended general improvements in lifestyle (91%), oral contraceptives (91%), metformin (85%), letrozole (74%), spironolactone (71%), specific diets (60%), medroxyprogesterone (45%), and anti-obesity medications (27%). Less than half of OB/GYNs (38%) reported referring patients with PCOS and obesity to other HCPs for treatment; these OB/GYNs estimated that on average they referred 25% of patients to registered dietitians/nutritionists, 20% to reproductive endocrinologists, 15% to general endocrinologists, 13% to primary care physicians, 8% to obesity medicine specialists and 7% to bariatric surgeons.

**FIG. 3.**
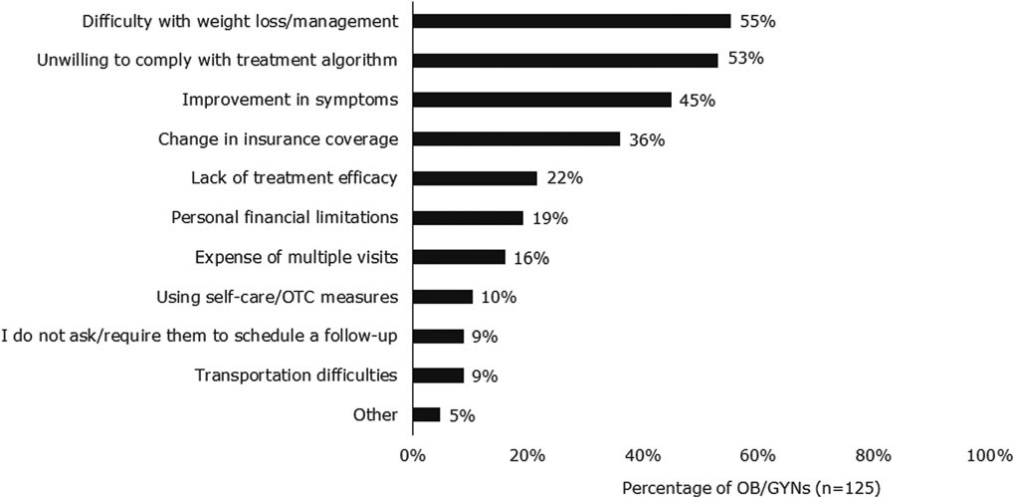
Reasons patients no longer seek care from OB/GYNs for PCOS. Data reported as frequency of OB/GYNs selecting each statement. OB/GYNs could select three reasons. OB/GYNs felt that difficulty with weight loss, unwillingness to comply with the treatment algorithm, and improvement in symptoms were the top reasons that patients no longer sought their care for PCOS. OTC, over the counter.

The OB/GYNs were asked for the top three reasons patients with PCOS and obesity no longer seek their care for PCOS management (Fig. 3). The most common responses were difficulty with weight management (55%), unwillingness to adhere to treatment algorithm (53%), and improvement in symptoms (45%). The top reasons patients gave for no longer seeing OB/GYNs for their PCOS care included moving away from the area (36%), not feeling they were helpful or necessary (22%), and change of insurance coverage (16%).

Most patients (65%) consider achieving sustained weight loss as one of the biggest challenges in managing their PCOS. OB/GYNs were most likely to schedule follow-up appointments quarterly (47%) or bi-annually (25%) for patients with PCOS and obesity after diagnosing them with PCOS. Almost all OB/GYNs said they conduct follow-up appointments themselves, rather than delegate follow-up appointments to nurse practitioners or physician assistants.

One third of patients said that OB/GYNs told them about weight loss diets or exercises (36%), helped them set weight loss goals (34%), helped them understand why they have excess weight (34%), or suggested they see a registered dietitian or nutritionist (31%). Fewer patients said that OB/GYNs discussed anti-obesity medications (22%) or referrals to weight management clinics (12%). Some patients (18%) said that their OB/GYNs never discussed weight management.

Almost all OB/GYNs (90%) believe that weight loss is necessary for PCOS symptom improvement compared with 72% of patients who believe that weight loss is necessary. OB/GYNs were asked what percentage of body weight a patient with PCOS and obesity would need to lose to improve PCOS symptoms. On average, OB/GYNs felt that patients would need to lose 16% (SD: 11) of their body weight to improve PCOS symptoms.

Very few OB/GYNs (10%) had received any formal obesity management training beyond medical school, but most (79%) were interested in receiving more. OB/GYNs were the least aware of obesity management guidelines and were significantly less likely (34%) than general endocrinologists (71%) and primary care physicians (53%) to feel highly confident in obesity management. Some OB/GYNs (30%) report that they refer patients with PCOS and obesity to bariatric surgeons.

**FIG. 4.**
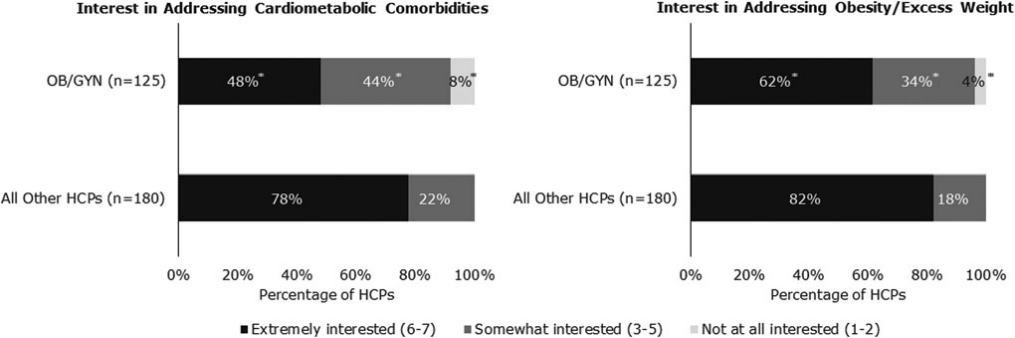
HCP interest in addressing patient comorbidities and obesity. Significantly fewer OB/GYNs were “extremely interested” or “somewhat interested” in addressing cardiometabolic comorbidities and obesity in their patients with PCOS and obesity when compared with other HCPs surveyed. Other HCPs surveyed included PCPs, general endocrinologists, and reproductive endocrinologists. *Indicates mean is significantly different from mean of all other HCPs (*p* < 0.05).

Almost all (98%) OB/GYNs understood that PCOS predisposes women to increased risk of developing cardiometabolic comorbidities. Compared with all other HCPs surveyed (PCPs, reproductive endocrinologists, and general endocrinologists), OB/GYNs expressed the least interest in addressing cardiometabolic comorbidities and excess weight (Fig. 4). However, more than half (62%) of OB/GYNs were extremely interested in addressing excess weight. Most OB/GYNs believed that consultations with a dietitian/nutritionist (75%) or access to a physician who specializes in obesity (67%) would be the most beneficial types of support for their patients with PCOS and obesity.

**FIG. 5.**
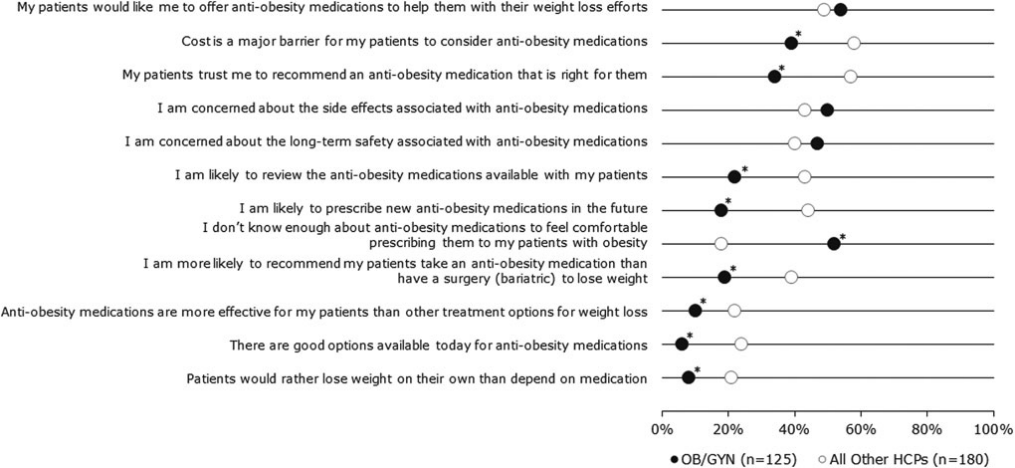
HCP attitudes to anti-obesity medications. The OB/GYN (black) attitudes to prescription anti-obesity medications compared with attitudes of all surveyed HCP (white). Shown as percentage of professionals “strongly agreeing (6/7)” or “completely agreeing (7/7)” with the listed statements. The OB/GYNs were significantly more likely than other HCPs to strongly agree that they do not know enough about anti-obesity medications to feel comfortable prescribing them to their patients with obesity. *Indicates that mean level of OB/GYN agreement differed significantly from mean level of agreement among all other HCPs surveyed (*p* < 0.05).

The OB/GYNs were asked to select the top three barriers to treating and managing obesity in their patients with PCOS. The top three barriers were lack of patient motivation/adherence (70%), lack of time during patient visit (59%), and lack of appropriate treatments (44%). The OB/GYNs were significantly more likely than other physicians surveyed to strongly agree with the statement that they do not know enough about anti-obesity medications to feel comfortable prescribing them to their patients with obesity (Fig. 5).

## Discussion

### Principal findings

OB/GYNs are the primary clinicians to diagnose and treat PCOS. OB/GYNs are most likely to prescribe lifestyle changes and oral contraceptives for patients with PCOS and obesity. Less than half of OB/GYNs refer patients with PCOS and obesity to other physicians for treatment. Both OB/GYNs and patients recognize obesity management as a major challenge to managing PCOS. Few OB/GYNs received obesity management training beyond medical school, but most express interest in receiving additional education on treating obesity.

### Results in the context of what is known

Previous survey research investigating patient experiences with PCOS has focused on health-related quality of life. Many surveys have reported that women were dissatisfied with their path to PCOS diagnosis, interactions with HCPs, and the efficacy of their PCOS care.^13,15,16^ A small qualitative survey in the United States found that women with PCOS experienced frustration with HCPs, confusion about the diagnostic process (long duration and number of HCPs seen) and lack of treatment plans, and a phase of searching for answers followed by gaining control.^16^

Our study came to similar conclusions with regards to patient frustrations with diagnosis and treatment of their PCOS. Our study investigated treatments used to manage patients' PCOS in greater detail compared to previous studies. We found that most OB/GYNs were prescribing lifestyle changes to manage PCOS in patients with PCOS and obesity, and patients saw managing their obesity as the biggest hurdle to successful treatment of their PCOS. Almost all OB/GYNs claimed to follow ACOG guidelines for the management of PCOS. The ACOG guidelines recommend exercise and dietary change to reduce risk of diabetes; insulin-sensitizing agents to reduce androgen levels, improve ovulation, and improve glucose tolerance; and letrozole for ovulation induction.^9^ The OB/GYNs in our study frequently recommended all of the above treatments for their patients with PCOS and obesity.

### Clinical implications

OB/GYNs cite a lack of education on obesity management and lack of time at appointments as major barriers preventing them from supporting patients' weight loss efforts. OB/GYNs also believed that their patients with PCOS and obesity would benefit from visits to registered dietitians/nutritionists or obesity specialists. Referrals to dietitians/nutritionists or obesity specialists may improve patient weight loss efforts. Small but statistically significant reductions in weight have been achieved with both dietitian/nutritionist support^17^ and behavioral interventions^18^ when compared with patients receiving the usual standard of care.

Bariatric surgery is an option that has produced stable long-term weight loss for patients with obesity.^19^ Approximately 30% of OB/GYNs in our study reported referring some of their patients with PCOS and obesity to bariatric surgeons. Less than half of OB/GYNs (38%) referred any patients with PCOS and obesity to other HCPs for treatment; on average, these OB/GYNs estimated that they referred 7% of patients with PCOS and obesity to bariatric surgeons. A meta-analysis of studies that investigated the impact of bariatric surgery on PCOS found significant decreases in menstrual irregularity and hirsutism at 12 months post-surgery and at study endpoint follow-up.^10^ Incidence of PCOS dropped from 45.6% pre-surgery to 6.8% (*p* < 0.001) at 12 months post-surgery.^10^

Compared with other HCPs surveyed, OB/GYNs in our study were less familiar and had less confidence prescribing anti-obesity medications. Federal Drug Administration-approved anti-obesity medications have demonstrated weight loss efficacy.^20,21^ Women with PCOS receiving orlistat (120 g) three times daily for three months had significant decreases in both weight and total testosterone.^22^ Orlistat has also been shown to improve BMI, waist circumference, insulin resistance, and lipid profiles in women with PCOS.^23–26^

A randomized clinical trial investigated the effect of treatment with exenatide (a glucagon-like peptide-1 receptor agonist [GLP-1 RA]), dapagliflozin (a sodium-glucose co-transporter 2 inhibitor [SGLT-2i]), exenatide/dapagliflozin, dapagliflozin/metformin extended-release, or phentermine/topiramate extended release on metabolism, body composition, and sex hormones in patients with PCOS and obesity.^27^ After 24 weeks of treatment, patients receiving exenatide/dapagliflozin and phentermine/topiramate experienced the greatest decreases in body weight and central adiposity.^27^

In addition, there is an ongoing clinical trial (the SAXAPCOS trial NCT03480022) investigating the effect of liraglutide (another GLP-1 RA) for treatment of patients with PCOS and obesity. These results suggest that medications targeting weight loss could play an important role in treating patients with PCOS and obesity.

### Research implications

Future work could investigate methods to improve obesity management in patients with PCOS and obesity. OB/GYNs may benefit from greater access to obesity management resources for their patients; these resources could include registered dietitians, anti-obesity medications, obesity specialists, and/or bariatric surgeons. Studies could investigate whether OB/GYNs with greater access to nurse practitioners or physician assistants and more frequent patient touchpoints have greater success managing patients with PCOS and obesity.

The Look AHEAD study found that patient attendance at scheduled intervention visits was strongly correlated with percentage weight loss at 1 year.^28^ OB/GYNs expressed an interest in receiving more education on obesity management. Other research confirms that obesity management is inadequately covered in medical school and residency.^29,30^ Future research will be needed to determine the best way to educate OB/GYNs about obesity management. Currently, OB/GYNs feel that obesity management is outside their scope of practice, but in the future, they could play an important role in supporting patient weight loss efforts.

### Strengths and limitations

Other studies highlight the confusion and delays that many women experience on their journey to a PCOS diagnosis and the frustrations they experience with lack of treatment efficacy.^15,16^ Our study provides a more quantitative analysis/evaluation of the typical patient journey and the types of treatments used. The cohort of patients with PCOS and obesity included may represent a particularly well-informed group of patients; however, we found that 4 in 10 women surveyed (42%) had an education at the high school level or equivalent, and an additional 22% had an associate degree (Table 1). This indicates a broader range of education attainment than is typically expected of online surveys.

Our study focuses on patients who appear to be highly engaged with PCOS care and may not be representative of patients who are still waiting on a PCOS diagnosis or who have chosen to self-manage their PCOS after disappointing experiences with HCPs.

The study patient population contains a smaller proportion of ethnic minorities than the U.S. population, which may limit generalizability to the broader population of women with PCOS in the United States. We did not collect residency information from patients, and it is possible that a patient's place of residence could impact their experience with PCOS care. Patients with a BMI <30 kg/m^2^ may experience longer delays in diagnosis with PCOS and are not included in this study.

Patients and physicians surveyed were not matched pairs, so some of the differences in responses between patients and physicians may represent real differences rather than differences in perception. Physicians included in this study were required to treat a minimum number of patients with PCOS and therefore represent a subset of physicians who are probably better informed about PCOS than the broader U.S. physician population.

## Conclusions

OB/GYNs are the primary HCPs responsible for treatment of patients with PCOS and obesity. OB/GYNs recognize the importance of weight loss for the treatment of PCOS but lack expertise in obesity management. OB/GYNs may benefit from more education on actionable weight loss strategies so that they can play a more active role in supporting obesity management in patients with PCOS and obesity. However, OB/GYNs identified capacity as a limiting factor in their ability to adequately support patients. Therefore, a multidisciplinary approach may be important to ensure that patients with PCOS and obesity receive the support they need in their weight loss efforts.

## Supplementary Material

Supplemental data

Supplemental data

## Data Availability

Data are available on request from the corresponding author.

## Supplementary Material

Supplementary Appendix Survey SA1

Supplementary Appendix Survey SA2
