# Supplementary material for: Polycystic Ovary Syndrome and Obesity: A Cross-Sectional Survey of Patients and Obstetricians/Gynecologists
Source: J Womens Health (Larchmt). 2023 Jun 6;32(6):723–31. doi: 10.1089/jwh.2022.0471 (PMC10278022; doi:10.1089/jwh.2022.0471)
Supplement: Supplemental data [file Supp_AppS1.pdf]

Appendix Survey A1. Patient survey.

PCOS Medical Interface Mapping Research – Questionnaire – Patients
October 7, 2020

Methodology:

- 25-minute online survey

Screening Criteria:

- Consents to terms
- Agrees to AE reporting
- Lives in the US
- 18-55 years old
- Born female and identifies as female
- Has PCOS
- Seeking help with fertility or other PCOS-related issue
- Has obesity (BMI ≥30)

SECTION S: SCREENER

ALL RESPONDENTS
S0 Thank you for your interest in this research. We appreciate your willingness to participate in this important research on healthcare issues. Before participating, KJT Group requires you to review the following information:

- KJT Group is a **global market research company**.
- Your responses to this survey will **help the sponsor design new products/services to meet patient needs**.
- Your responses will be kept **strictly confidential** and will never be associated with your name (double-blind).
- We expect, on average, it will take respondents like yourself **25 minutes** to complete this survey.
- Your **participation is voluntary**, and you may choose to stop participating at any time (withdraw consent).

If you qualify for and complete the survey, you will be eligible to receive the honorarium referenced in your invitation.

Do you consent to these terms and wish to continue?

1. Yes **CONTINUE**
2. No **TERMINATE**

[IF CONSENTS TO TERMS (S0r1) ASK AE1. ELSE TERMINATE]

CONSENTS TO TERMS (S0r1)
AE1 We are required to pass on to the pharmaceutical company sponsoring the study details of adverse events and/or other safety information - hereinafter referred to as safety information - that are mentioned during this study. Although what you say will be treated in confidence, should you mention safety information during the study, we will need to report it even if you have already reported it to the company or regulatory authorities.

In relation to reporting safety information, situation we need to know if you are willing to waive the confidentiality given to you under the Market Research Codes of Conduct. In the event that you waive confidentiality in relation to safety information reporting, any personal data provided during the reporting will be processed as follows:

a) Any personal data in relation to the safety information reported will be forwarded to the project sponsor; and

b) The project sponsor will record any safety information, including personal data received in the sponsor's global database, in the interests of patient safety and in compliance with all applicable global laws and regulations; and

c) During the reporting of safety information, the project sponsor will not disclose such personal data to any un-associated third parties, with the exception of any disclosures required by applicable law, regulation or the order of a competent authority.

Do you agree to waive the confidentiality given to you under the Market Research Codes of Conduct in relation to any safety information you report to us? If you agree, your contact details will be forwarded to the sponsor's Safety department for the express and sole purpose of follow-up of such report(s). Details of safety information maybe reported to regulatory authorities along with your personal data. All other information provided by you in this study will remain confidential. If you prefer to preserve the confidentiality of this information, please select 'I do not agree'. If you do so, you can still participate in this survey.

1. I agree   Please enter your email here: [OPEN TEXT BOX]
2. I do not agree

[IF AGREES (AE1r1) READ AE2. IF DISAGREES (AE1r2) ASK AE3]

AGREES (AE1r1)
AE2 Thank you. Please note that if your email address is provided during the Adverse Event, other safety information or product complaints reporting, this will not be linked in any way to your responses given during the interview.

Are you happy to proceed with this research?

1. Yes **CONTINUE**
2. No **TERMINATE**

[IF YES (AE2r1) JUMP TO S1; ELSE TERMINATE]

DOES NOT AGREE (AE1r2)
AE3 If we become aware of safety information, we are obliged to report this to the pharmaceutical company. We will file this report without giving any of your details.

Are you happy to proceed with this research?

1. Yes **CONTINUE**
2. No **TERMINATE**

[IF HAPPY TO PROCEED (AE3r1) ASK S1. ELSE TERMINATE]

AGREES TO AE REPORTING (AE2r1 OR AE3r1)
S1 In what state is your residence?

If you have residences in more than one state, please select the state where you spend the majority of the time.

[INSERT STATE DROP DOWN] **TERMINATE IF NOT U.S.**

[IF LIVES IN THE U.S. (S10r1=US STATE) ASK S1A. ELSE TERMINATE]

LIVES IN THE U.S. (S10r1=US STATE)
S1A HIDDEN QUESTION FOR REGION

1. Northeast
   [S1=CT, MA, ME, NH, NJ, NY, PA, RI, VT]
2. Midwest
   [S1=IA, IL, IN, KS, MI, MN, MO, ND, NE, OH, SD, WI]
3. South
   [S1=AL, AR, DC, DE, FL, GA, KY, LA, MD, MS, NC, OK, SC, TN, TX, VA, WV]
4. West
   [S1=AK, AZ, CA, CO, HI, ID, MT, NM, NV, OR, UT, WA, WY]

LIVES IN THE U.S. (S1r1=US)
S2 In what year were you born?

Please enter as a four-digit number, e.g., 1963

[RANGE: 1890-2019]
|_|_|_|_|

LIVES IN THE U.S. (S1r1=US STATE)
S2A HIDDEN COMPUTE FOR AGE **CONTINUE IF 18+ (CONTINUE BUT MARK AS NOT QUALIFIED IF >55)**

[IF 18-55 YEARS OLD (S2Ar1=18-55) ASK S3; IF 56+ MARK AS NOT QUALIFIED AND CONTINUE; ELSE TERMINATE IMMEDIATELY]

18+ YEARS OLD (S2Ar1=18+)
S3 Are you…?

Please select all that apply.

[MULTIPLE SELECT]
1. White
2. Black or African American
3. Spanish/Hispanic or Latino
4. American Indian or Alaska Native
5. Asian
6. Middle Eastern
96. Other, please specify: ________
97. Decline to answer [EXCLUSIVE]

18+ YEARS OLD (S2Ar1=18+)
S4 What sex were you assigned at birth, on your original birth certificate?

1. Male **TERMINATE**
2. Female **CONTINUE**

[IF BORN FEMALE (S4r2) ASK S4. ELSE TERMINATE]

BORN FEMALE (S4r2)
S5 How do you describe yourself?

1. Male **TERMINATE**
2. Female **CONTINUE**
3. Transgender **TERMINATE**
4. Do not identify as female, male or transgender **TERMINATE**

[IF SELF IDENTIFY AS FEMALE (S5r2) ASK S10. ELSE TERMINATE]

IDENTIFY AS FEMALE (S5r2)

S10 With which, if any, of the following conditions have you ever been diagnosed by a healthcare professional (physician, nurse practitioner, etc.)?

Please select all that apply.

[RANDOMIZE; GROUP CODES 1/2/3/4/5, 6/7, 8/9/10/11, 14/15]

1. PCOS (Polycystic ovary syndrome) **CONTINUE (MARK AS NOT QUALIFIED IF THIS IS NOT SELECTED)**
2. Infertility **CONTINUE**
3. Hyperandrogenism **CONTINUE**
4. Anovulation or oligo-anovulation (missed or irregular ovulation) **CONTINUE**
5. Hirsutism (excessive hair growth) **CONTINUE**
6. Pre-diabetes (which is a slightly elevated blood glucose levels, regarded as indicative that a person is at risk of progressing to Type 2 diabetes)
7. Type 2 Diabetes
8. Cardiovascular / heart disease
9. Hypertension (high blood pressure)
10. Dyslipidemia (high cholesterol or high triglycerides)
11. Non-Alcoholic Fatty Liver Disease (NAFLD) / Non-alcoholic steatohepatitis (NASH)
12. Cancer
13. Obesity
14. Depression
15. Anxiety
99. None of the above [EXCLUSIVE]

[IF HAS PCOS OR RELATED CONDITIONS (S10r1-5 ANY) ASK S15; ELSE TERMINATE]

HAS PCOS OR RELATED CONDITIONS (S10r1-5)
S15 Which, if any, of the following currently apply to you?

I would like help with…

[RANDOMIZE]

1. Fertility **CONTINUE**
2. Reducing excessive hair growth (hirsutism) **CONTINUE**
3. Irregular or missed periods **CONTINUE**
4. Losing weight **CONTINUE**
99. None of the above [EXCLUSIVE] **CONTINUE BUT MARK AS NOT QUALIFIED**

[IF NOT CURRENTLY SEEKING HELP WITH FERTILITY (DID NOT SELECT S15r1) ASK S17; ELSE JUMP TO S20A]

NOT CURRENTLY SEEKING HELP WITH FERTILITY (DID NOT SELECT S15r1)
S17 Do you want to try to become pregnant in the future?

1. Yes
2. No
3. I am not sure

[IF DESIRES FUTURE PREGNANCY (S17r1) ASK S17A. EKSE SKIP TO S20A]

DESIRES FUTURE PREGNANCY (S17R1)
S17A Do you plan to try to become pregnant within the next year?

1. Yes
2. No
3. I am not sure

HAS PCOS OR RELATED CONDITIONS (S10r1-5)
S20A What is your height (feet, inches)?

Your best estimate will do.

[RANGE 1-9]
Feet: |_| and
[RANGE 0-11]
Inches: |_|_|

HAS PCOS OR RELATED CONDITIONS (S10r1-5)
S20B What is your current weight (pounds)?

Please be as exact as possible.

[RANGE 50-1000]
Pounds |_|_|_|_|

HAS PCOS OR RELATED SYMPTOMS (S10r1-5)
S20C HIDDEN QUESTION FOR BMI CALCULATION

[BMI = (S20Br1*703) / (S20Ar1*12 + S20Ar2) ^2]

HAS PCOS OR RELATED SYMPTOMS (S10r1-5)
S20D HIDDEN QUESTION FOR WEIGHT CLASSIFICATION

1. Underweight (S20C < 18.5)
2. Normal Range (S20C ≥ 18.5 AND S20C < 25)
3. Overweight (S20C ≥ 25 AND S20C < 30)
4. Obese Class I (S20C ≥ 30 AND S20C < 35)
5. Obese Class II (S20C ≥ 35 AND S20C < 40)
6. Obese Class III (S20C ≥ 40)

ALL RESPONDENTS
S100 HIDDEN QUOTA QUESTION

1. **QUALIFIED PATIENT WITH PCOS [N=300]**
    - CONSENTS TO TERMS (S0r1)
    - AGREES TO AE REPORTING (AE2r1 OR AE3r1)
    - LIVES IN THE US (S1r1=US STATE)

- 18-55 YEARS OLD (S2Ar1=18-55)
- BORN FEMALE (S4r2)
- IDENTIFY AS FEMALE (S5r2)
- HAS PCOS (S10r1)
- SEEKING HELP WITH FERTILITY OR OTHER PCOS RELATED ISSUE (S15r1-4)
- HAS OBESITY (BMI ≥30; S20Dr4-6)

99. NOT QUALIFIED [N=999]

ALL QUALIFIED RESPONDENTS (S100r1)
S105 FERTILITY ASSISTANCE QUOTA

1. Currently seeking fertility assistance or desiring pregnancy in the next year (S15r1 OR (S17r1 AND S17Ar1) [N=50]
2. Not seeking help but desires pregnancy but not within the next year (S17r1 AND S17Ar2-3) [N=125]
3. Not seeking help and does not desire pregnancy in the future/not sure (S17r2-3) [N=125]

ALL QUALIFIED RESPONDENTS (S100r1)
S110 SOFT QUOTA AGE

1. 18-25 years old (S2Ar1=18-25) [N=999]
2. 26-40 years old (S2Ar1=26-40) [N=999]
3. 41-55 years old (S2Ar1=41-55) [N=75]

ALL QUALIFIED RESPONDENTS (S100r1)
S115 SOFT QUOTA BMI

1. Overweight (S20Dr3) [N=0]
2. Obesity Class I (S20Dr4) [N=999]
3. Obesity Class II (S20Dr5) [N=999]
4. Obesity Class III (S20Dr6) [N=999]

ALL QUALIFIED RESPONDENTS (S100r1)
S120 SOFT QUOTA ETHNICITY

[MULTIPLE SELECT; MAX OF N=200 RESPONDENTS WHO ARE ONLY CODE 1]

1. White (S3r1) [N=999]
2. Black or African American (S3r2) [N=999]
3. Middle Eastern (S3r6) [N=999]
4. Spanish/Hispanic (S3r3) [N=999]
96. Other (S3r4-5,96) [N=999]
97. Decline to answer (S3r97) [N=999]

SECTION 200: PRE-DIAGNOSIS

ALL QUALIFIED RESPONDENTS (S100r1)
Q200 You have qualified for the full survey. Thank you for your responses thus far. The remainder of this survey should take approximately 22 minutes to complete. As a reminder, your responses to this survey are critical to the success of this research in helping the sponsor design new products/services to help you support your needs. Your responses will be kept strictly confidential and only reported in combination with other respondents' data. In addition, you may be asked certain questions for quality control purposes.

ALL QUALIFIED RESPONDENTS (S100r1)
Q203 For the remainder of the survey, we would like to understand your experiences being diagnosed with, and treated for, polycystic ovary syndrome **— referred to as PCOS.** We will ask about the time from when you first started experiencing symptoms until today.

To the best of your knowledge, which of the following are the cause(s) of PCOS?

Please select all that apply.

[RANDOMIZE, MULTIPLE SELECT]
1. Hormonal imbalance
2. Excess weight / being overweight
3. Genetics
4. Another condition I have been diagnosed with
5. Insulin resistance
6. Lifestyle habits (i.e., diet, exercise)
96. Other, please specify: [INSERT TEXT BOX. ANCHOR]
97. Not sure [EXCLUSIVE, ANCHOR]

ALL QUALIFIED RESPONDENTS (S100r1)
Q208 What PCOS symptoms did you experience prior to discussing your symptoms for the first time with a healthcare provider?

Please select all that apply.

[MULTPLE SELECT, RANDOMIZE]
1. Weight gain
2. Irregular periods
3. Excessive hair growth
4. Acne
5. Painful periods
6. Low fertility / Inability to get pregnant
7. Absence of periods
8. Thinning hair
9. Ovarian cysts
96. Other, please specify: [ANCHOR, MANDATORY TEXT BOX]
97. I didn't experience (or didn't realize I was experiencing) PCOS symptoms until my provider mentioned them [EXCLUSIVE][ANCHOR]

ALL QUALIFIED RESPONDENTS (S100r1)
Q208A At what age did you first experience PCOS symptom(s)?

|_|_| years old

ALL QUALIFIED RESPONDENTS (S100r1)
Q210 Before talking to a healthcare provider about your symptoms, what, if anything did you do to manage your symptoms on your own, regardless of whether or not you attributed these symptoms to PCOS at the time?

Please select all that apply.

To manage my symptoms, I…

[RANDOMIZE, MULTIPLE SELECT; GROUP CODES 6/7]
1. Used over-the-counter treatments for menstrual pain (e.g., Midol, Advil, Tylenol, herbal supplements)
2. Used over-the-counter treatments for excessive hair growth (e.g., Nair, herbal supplements)
3. Used over-the-counter treatments for acne (e.g., facial scrubs, Proactiv)
4. Used over-the-counter treatments for weight loss (e.g., Hydroxycut, herbal supplements such as green coffee bean, green tea)
5. Used over-the-counter treatments for thinning hair (e.g., Rogaine, herbal supplements such as biotin)
6. Reduced my physical activity
7. Increased my physical activity
8. Changed my diet

96. Other, please specify: [INSERT TEXT BOX][ANCHOR]
97. I did not do anything to manage my symptoms on my own [EXCLUSIVE; ANCHOR]

ALL QUALIFIED RESPONDENTS (S100r1)
Q216 What caused you to discuss your PCOS symptoms with a healthcare provider?

Please select all that apply.

[RANDOMIZE; GROUP CODES 6/7 MULTIPLE SELECT]
1. My symptoms worsened (in degree of discomfort/frequency/etc.)
2. My symptoms were interfering too much with my daily life
3. My symptoms were not resolving on their own
4. I was encouraged by family or friends
5. I had trouble getting pregnant or experienced pregnancy-related complications (e.g., miscarriage)
6. I started experiencing new symptoms that concerned me
7. My symptoms required immediate attention (e.g., ruptured cysts)
8. My healthcare provider asked me about my symptoms

96. Other, please specify: [ANCHOR. INSERT TEXT BOX]

SYMPTOMS INTERFERED WITH DAILY LIFE (Q216r2)
Q216A You indicated that one of the reasons you discussed your PCOS symptoms with a healthcare provider was your symptoms were interfering too much with your daily life.

Which of the following symptoms were interfering with your daily life?

Please select all that apply.

[MULTPLE SELECT, RANDOMIZE. SHOW ONLY THOSE SELECTED AT Q208]

1. Weight gain
2. Irregular periods
3. Excessive hair growth
4. Acne
5. Painful periods
6. Low fertility / Inability to get pregnant
7. Absence of periods
8. Thinning hair
9. Ovarian cysts

98. [Q208r96 RESPONSE]

ALL QUALIFIED RESPONDENTS (S100r1)

Q220 For what reasons did you not bring up your symptoms with your healthcare provider sooner?

Please select all that apply.

[RANDOMIZE. MULTIPLE SELECT]

1. I didn't want to admit I was experiencing a health issue
2. I didn't think there was anything that my healthcare provider could do about it
3. I was afraid to find out what was going on
4. I thought my symptoms were due to normal puberty or heredity
5. I thought my symptoms were due to another health condition
6. I was able to manage my symptoms with self-care methods (e.g., over-the-counter treatments, a change in physical activity or diet)
7. My symptoms did not limit my daily function and activities up until that point

96. Other, please specify: [ANCHOR. INSERT TEXT BOX]
97. I didn't wait to bring up my symptoms – it was the soonest I could get an appointment [EXCLUSIVE; ANCHOR]

ALL QUALIFIED RESPONDENTS (S100r1)

Q225 Prior to being formally diagnosed with PCOS by a healthcare provider, how many healthcare providers did you see specifically because of your PCOS symptoms?

If you were diagnosed with PCOS at the first visit related to your PCOS symptoms, please enter "0."

1. |_|_| healthcare providers

ALL QUALIFIED RESPONDENTS (S100r1)

Q230 Prior to being formally diagnosed with PCOS by a healthcare provider, were you misdiagnosed with another condition due to your PCOS symptoms?

1. Yes
2. No

MISDIAGNOSED (Q230r1)

Q235 What type(s) of healthcare providers misdiagnosed you?

Please select all that apply.

[RANDOMIZE. MULTISELECT]

1. Primary Care Provider
2. OB/GYN
3. Bariatric Surgeon
4. Obesity Medicine Specialist
5. General Endocrinologist
6. Reproductive Endocrinologist
7. Registered Dietitian / Nutritionist
96. Other, please specify: [ANCHOR. INSERT TEXT BOX]

MISDIAGNOSED (Q230r1)
Q235A What condition(s) were you misdiagnosed with?

[ROWS]
[PIPE IN PROVIDERS SELECTED AT Q235]

[COLUMN HEADER: "Condition(s) diagnosed with"]

[MANDATORY TEXT BOX]

SECTION 600: PATHWAY

ALL QUALIFIED RESPONDENTS (S100r1)
Q207 You mentioned that you first started experiencing PCOS symptoms at [PIPE Q208Ar1] years old.

How long after you first started experiencing symptoms at age [PIPE Q208Ar1] did you **first discuss your symptoms with a healthcare provider**?

If less than one year, enter 0 for "years" and then only enter in the number of months. If less than one month, enter "0" for both "months" and "years."

|_|_| years [RANGE: 0-(S3-Q208A)]
|_|_| months [RANGE: 0-11]

ALL QUALIFIED RESPONDENTS (S100/1)
Q207B BTS MONTHS CALCULATION

[INSERT MONTHS: Q207r1/12 + Q207r2]

ALL QUALIFIED RESPONDENTS (S100r1)
Q207C You mentioned that you first started experiencing PCOS symptoms at [PIPE Q208Ar1] years old.

How long after you first started experiencing symptoms at age [PIPE Q208Ar1] were you **formally diagnosed with PCOS by a healthcare provider?**

If less than one year, enter 0 for "years" and then only enter in the number of months. If less than one month, enter "0" for both "months" and "years."

|_|_| years [RANGE: 0-(S3-Q206)]
|_|_| months [RANGE: 0-11]

ALL QUALIFIED RESPONDENTS (S100r1)
Q207D BTS MONTHS CALCULATION

[INSERT MONTHS: Q207Cr1/12 + Q207Cr2]

ALL QUALIFIED RESPONDENTS (S100r1)
Q207E You mentioned that you first started experiencing PCOS symptoms at [PIPE Q208Ar1] years old.

How long after you first started experiencing symptoms at age [PIPE Q208Ar1] were you **first treated specifically for PCOS?**

If less than one year, enter 0 for "years" and then only enter in the number of months. If less than one month, enter "0" for both "months" and "years."

|_|_| years [RANGE: 0-(S3-Q206)]
|_|_| months [RANGE: 0-11]

ALL QUALIFIED RESPONDENTS (S100r1)
Q207F BTS MONTHS CALCULATION

[INSERT MONTHS: Q207Er1/12 + Q207Er2]

ALL QUALIFIED RESPONDENTS (S100r1)
Q600 Now, we want to know more about the types of healthcare providers you've seen for PCOS over time **-** from the <u>first discussion of symptoms until today</u>.

What specialties of healthcare providers (or the specialty of their office/clinic) have you **ever** seen for PCOS?

Please consider all providers you saw (regardless of the initial reason) so long as seeing them was related to PCOS (i.e., initial evaluation, diagnosis, ongoing treatment and management, weight management related to PCOS).

[RANDOMIZE, GROUP CODES 3/4; MULTIPLE SELECT]
1. Primary Care
2. OB/GYN
3. Bariatric Surgery
4. Obesity Medicine
5. General Endocrinology
6. Reproductive Endocrinology
7. Nutrition (i.e., Registered Dietitian/Nutritionist)
96. Other, please specify: [MANDATORY TEXT BOX. ANCHOR]

ALL QUALIFIED RESPONDENTS (S100r1)
Q602 Do you remember what type of healthcare provider first told you your symptoms <u>might be PCOS</u> (i.e., used the term "PCOS" or said they suspected you had PCOS), <u>regardless</u> of whether they confirmed your diagnosis?

1. Yes
2. No

REMEMBERS PROVIDER (Q602r1)
Q602A What specialty of healthcare provider first told you your symptoms might be PCOS (i.e., used the term "PCOS" or said they suspected you had PCOS), regardless of whether they confirmed your diagnosis?

[RANDOMIZE, GROUP CODES 3/4]
1. Primary Care
2. OB/GYN
3. Bariatric Surgery
4. Obesity Medicine
5. General Endocrinology
6. Reproductive Endocrinology
7. Nutrition (i.e., Registered Dietitian/Nutritionist)
96. Other, please specify: [MANDATORY TEXT BOX. ANCHOR]

ALL QUALIFIED RESPONDENTS (S100r1)
Q605 How many of each of the following specialties of provider have you **ever** seen for PCOS?

Again, please consider all providers you saw (regardless of the reason) so long as seeing them was related to PCOS (i.e., initial evaluation, diagnosis, ongoing treatment and management, weight management related to PCOS).

[COLUMN]
1. Number of providers

[ROWS, ONLY SHOW THOSE SELECTED AT Q600 IN SAME ORDER; RANGE 1-99]
1. Primary Care |_|_|_|
2. OB/GYN |_|_|_|
3. Bariatric Surgery |_|_|_|
4. Obesity Medicine |_|_|_|
5. General Endocrinology |_|_|_|
6. Reproductive Endocrinology |_|_|_|
7. Nutrition (i.e., Registered Dietitian/Nutritionist) |_|_|_|
96. [INSERT Q600r96 RESPONSE] [MANDATORY TEXT BOX. ANCHOR]

ALL QUALIFIED RESPONDENTS (S100r1)
Q605A HIDDEN QUESTION – NUMBER OF PROVIDER TYPES SEEN

[SELECT PROVIDERS BELOW BASED ON NUMERIC ENTRY AT Q605]
1. Primary Care Provider 1
2. Primary Care Provider 2
3. OB/GYN 1
4. OB/GYN 2
5. Bariatric Surgeon 1
6. Bariatric Surgeon 2
7. Obesity Medicine Specialist 1
8. Obesity Medicine Specialist 2
9. General Endocrinologist 1
10. General Endocrinologist 2
11. Reproductive Endocrinologist 1
12. Reproductive Endocrinologist 2
13. Nutritionist 1
14. Nutritionist 2

15. [INSERT Q600r96 RESPONSE] 1
16. [INSERT Q600r96 RESPONSE] 2

ALL QUALIFIED RESPONDENTS (S100r1)
Q605B Now, for each of the following providers, please enter their initials – we will refer to providers in the questions that follow using their initials. For example, Dr. Anne Smith would be "A.S." If more than one provider has the same initials, please make a unique identifier to differentiate – for example, "A. Smith."

Please note: from this point on we will only ask you about up to 2 providers of a particular specialty, even if you have seen more than 2. If you've seen more than 2:

- ***Provider 1 should be the first provider*** *you saw of a particular specialty*
- ***Provider 2 should be*** *another provider* ***– whichever has been most influential on your PCOS care****.*

For example, if you saw three OB/GYNs, you would enter the initials of the 1st OB/GYN you saw for PCOS as OB/GYN 1, and for OB/GYN 2 you would enter the initials of one of the other two OB/GYNs you've seen – whichever has been most influential on your PCOS care.

[COLUMNS]

1. Initials (or other unique identifier) |_|_|_|

[ROWS; INSERT BASED ON Q605A]

1. Primary Care Provider 1
2. Primary Care Provider 2
3. OB/GYN 1
4. OB/GYN 2
5. Bariatric Surgeon 1
6. Bariatric Surgeon 2
7. Obesity Medicine Specialist 1
8. Obesity Medicine Specialist 2
9. General Endocrinologist 1
10. General Endocrinologist 2
11. Reproductive Endocrinologist 1
12. Reproductive Endocrinologist 2
13. Nutritionist 1
14. Nutritionist 2
15. [INSERT Q600r96 RESPONSE] 1
16. [INSERT Q600r96 RESPONSE] 2

[FOR QUESTIONS BELOW REQUIRING PIPING OF PROVIDER, DISPLAY AS ROW LABEL AND INITIALS FROM Q605Bc1 IN PARENTHESES, BUT CODE AS ROW LABEL]

ALL QUALIFIED RESPONDENTS (S100r1)
Q610 Now, please place the providers you have seen in chronological order starting with the type of healthcare provider you saw first, the type you saw next, etc.

If you indicated you saw more than one of a provider type, multiple options for each provider type will be available – for example, if you saw two OB/GYNs, "OB/GYN 1" will be the first OB/GYN you saw. If you saw more than two of a provider type, please use the 2nd provider category to indicate the most recent provider of that type seen.

[SHOW RELEVANT SELECTION FROM Q605A USING ROW LABEL AND INITIALS FROM Q605B IN PARENTHESES. DRAG AND DROP RANK ORDER]

ALL QUALIFIED RESPONDENTS (S100r1)
Q615 For what reason(s) have you ever seen the following providers related to your PCOS care?

Please select all that apply.

You may only select one provider for "diagnosis" and "initial treatment."

[FORCE RESPONSE FOR EACH ROW. FORCE RESPONSE FOR C2 AND C4, DO NOT FORCE RESPONSE FOR ALL OTHER COLUMNS]

[COLUMNS]

1. Initial symptom evaluation (prior to diagnosis)
2. Diagnosis of PCOS [ONLY ALLOW 1 SELECTION]
3. Second opinion for diagnosis
4. Initial treatment for PCOS symptoms [ONLY ALLOW 1 SELECTION]
5. Ongoing treatment and management of PCOS
6. Weight management related to PCOS

[ROWS]
[SHOW SELECTIONS FROM Q605A IN Q610 ORDER WITH ROW LABEL AND Q605B INITIALS IN PARENTHESES]

[LOOP Q640A-Q670 FOR ALL PROVIDERS SEEN AT Q605A IN Q610 ORDER WITH ROW LABEL AND Q605B INITIALS IN PARENTHESES]

ALL QUALIFIED RESPONDENTS (S100r1)
Q640A Is [INSERT ROW LABEL (INSERT Q605B PROVIDER INITIALS)] a physician?

By physician we mean a medical doctor (MD) rather than a nurse practitioner (NP) or physician assistant (NP)

1. Yes
2. No
3. I am not sure / do not recall

ALL QUALIFIED RESPONDENTS (S100r1)
Q665 Are you still seeing [INSERT ROW LABEL (INSERT Q605B PROVIDER INITIALS)]?

1. Yes
2. No

DOESN'T STILL SEE HCP (Q665r2)
Q670 For what reasons do you no longer see [INSERT ROW LABEL (INSERT Q605B PROVIDER INITIALS)]?

Please select all that apply.

[RANDOMIZE. MULTIPLE SELECT]
1. I moved away from the area they are in

2. Did not feel they were helpful/necessary
3. No follow-up visit was scheduled / recommended by provider
4. They did all they could to help, but it wasn't enough
5. Started seeing another type of provider
6. My insurance coverage changed
96. Other, please specify: [INSERT TEXT BOX, ANCHOR]

[LOOP FOR ALL Q605A PROVIDERS IN Q610 ORDER]

[END OF LOOP]

ALL QUALIFIED RESPONDENTS (S100r1)
Q675 Now, we'd like to learn a little more about the treatments you have used for PCOS.

Which of the following treatments have you **ever** been prescribed or recommended by a healthcare provider and used for treatment of your PCOS symptoms, and which are you **currently** using?

[COLUMNS]
1. I am currently using this treatment
2. I used this treatment in the past, but I am not currently using it
3. I have never used this treatment

[ROWS. MULTIPLE SELECT. RANDOMIZE] [ONLY ALLOW ONE SELECTION PER ROW]
1. Over the counter treatments for PCOS symptoms, such as menstrual pain, acne, excessive hair growth (e.g., Midol, Proactiv, Nair)
2. Over-the-counter treatments for weight loss (e.g., Hydroxycut, herbal supplements such as green coffee bean, green tea)
3. Oral contraceptives (birth control pills)
4. Metformin (Glucophage, Riomet)
5. Letrozole (Femara) / Clomid (Clomiphene)
6. Spironolactone (CaroSpir, Aldactone)
7. Provera (Depo-Provera)
8. Lifestyle changes (e.g., improving eating habits, increasing physical activity, using a specific diet or diet program (Jenny Craig, Weight Watchers, Elimination diets, Medifast))
9. Prescription weight loss medication (e.g., Saxenda, Xenical, Qsymia, Contrave)
96. Other, please specify: [INSERT TEXT BOX. ANCHOR]
97. I have never been treated for my PCOS symptoms [ANCHOR; EXCLUSIVE]

[IF HAS EVER BEEN TREATED FOR PCOS (Q675r1-96) ASK Q677. ELSE JUMP TO Q306.]

HAS EVER BEEN TREATED FOR PCOS (Q675r1-96)
Q677 Which of the following treatments prescribed or recommended by a healthcare provider did you use prior to your diagnosis of PCOS?

Please only consider treatment initiated or suggested by a healthcare provider.

Please select all that apply.

[ROWS. MULTIPLE SELECT. RANDOMIZE. ONLY SHOW TREATMENTS SELECTED AT Q675c1-2]

1. Over the counter treatments for PCOS symptoms, such as menstrual pain, acne, excessive hair growth (e.g., Midol, Proactiv, Nair)
2. Over-the-counter treatments for weight gain (e.g., Hydroxycut, herbal supplements such as green coffee bean, green tea)
3. Oral contraceptives (birth control pills)
4. Metformin (Glucophage, Riomet)
5. Letrozole (Femara) / Clomid (Clomiphene)
6. Spironolactone (CaroSpir, Aldactone)
7. Provera (Depo-Provera)
8. Lifestyle changes (e.g., improving eating habits, increasing physical activity, using a specific diet or diet program (Jenny Craig, Weight Watchers, Elimination diets, Medifast))Prescription weight loss medication (e.g., Saxenda, Xenical, Qsymia, Contrave)

96. Other, please specify: [INSERT TEXT BOX. ANCHOR]
97. I was not treated for my PCOS symptoms prior to diagnosis of PCOS [ANCHOR; EXCLUSIVE]

ALL QUALIFIED RESPONDENTS (S100r1)
QC2 For quality control purposes, please select “slightly happy” from the list of options below.

1. Very unhappy
2. Slightly unhappy
3. Neutral
4. Slightly happy
5. Very happy

SECTION 300: DIAGNOSIS

ALL QUALIFIED RESPONDENTS (S100r1)
Q306 Now that we understand more about your experience with PCOS overall, we want to know more about some specific points in time related to your diagnosis and ongoing care. First, we want to learn a little more about your experiences **being diagnosed with PCOS**.

Which of the following best describes how you were formally diagnosed with PCOS?

1. I was diagnosed at a well-visit or annual exam
2. I was diagnosed at an appointment made specifically to discuss my PCOS symptoms
3. I was diagnosed at an appointment for another condition

96. Other, please specify: [MANDATORY TEXT BOX][ANCHOR]
97. I do not recall [ANCHOR]

ALL QUALIFIED RESPONDENTS (S100r1)
Q310 Which of the following did your **diagnosing provider** use to formally diagnose you with PCOS?

Please select all that apply.

[MULTIPLE SELECT. RANDOMIZE.]

1. Blood testing
2. Pelvic exam
3. Ultrasound
4. My description of my symptoms

96. Other, please specify: [INSERT TEXT BOX. ANCHOR]
97. I am not sure/do not recall [EXCLUSIVE][ANCHOR]

ALL QUALIFIED RESPONDENTS (S100r1)
Q316 When you were first diagnosed with PCOS, which of the following did your **diagnosing provider** talk to you about?

Please select all that apply.

[RANDOMIZE; MULTIPLE SELECT]
1. Causes of PCOS
2. Treatments for PCOS (e.g., lifestyle modifications, prescription medications)
3. How PCOS is related to, or impacts, my other health conditions
4. Progression of PCOS (what to expect in the future)
96. Other, please specify: [INSERT TEXT BOX. ANCHOR]
97. I do not recall [EXCLUSIVE. ANCHOR]
98. None of these [EXCLUSIVE. ANCHOR]

ALL QUALIFIED RESPONDENTS (S100r1)
Q320 When you were first diagnosed with PCOS, what, if any, resources did your **diagnosing provider** provide to help with your understanding of PCOS or how to manage/treat it?

Please select all that apply.

[MULTIPLE SELECT]
1. Reading material (i.e., pamphlet/brochure, book, magazine) about the condition
2. Website
3. Information on local support group
4. Smartphone app
96. Other, please specify: [INSERT TEXT BOX. ANCHOR]
97. My healthcare provider did not provide me with any resources at this time [EXCLUSIVE. ANCHOR]
98. I do not recall [EXCLUSIVE. ANCHOR]

ALL QUALIFIED RESPONDENTS (S100r1)
Q326 When you were first diagnosed with PCOS, did your **diagnosing provider** specifically discuss your weight or weight management with you?

1. Yes
2. No
3. I don’t recall

[IF DISCUSSED WEIGHT (Q326r1) ASK Q330. ELSE SKIP TO Q400A]

DISCUSSED WEIGHT (Q326r1)
Q330 When you were first diagnosed with PCOS, which, if any, of the following did your **diagnosing provider** discuss about your weight?

Please select all that apply. Please consider only the things your healthcare provider did/discussed when you were initially diagnosed, not anything that they may have recommended later in your care.

[MULTIPLE SELECT. RANDOMIZE. GROUP CODES 1/2]
1. Explain the effect my weight has on my PCOS
2. Explain the effect my PCOS has on my weight
3. Help me set goals to improve my weight

4. Help me understand why I have excess weight
5. Make me aware of medications that will help me lose weight
6. Tell me about ways to lose weight (i.e., programs [e.g., Jenny Craig], diet, exercise)
7. Tell me about weight loss clinics
8. Suggest I see a registered dietitian or nutritionist
96. Other, please specify: [INSERT TEXT BOX. ANCHOR]
97. I do not recall [ANCHOR. EXCLUSIVE]

SECTION 400: ONGOING MANAGEMENT

ALL QUALIFIED RESPONDENTS (S100r1)
Q400A Now, we'd like to learn a little more about the ongoing treatment and management of your PCOS.

Please click to continue.

ALL QUALIFIED RESPONDENTS (S100r1)
Q405A Below are the providers you have seen for PCOS treatment and management. Please select the two providers that have been **most influential** in the treatment and management of your PCOS.

Please select two.

[RANDOMIZE; IF ONLY ONE RESPONSE AVAILABLE AUTOFILL AND SKIP]

1. [SHOW SELECTIONS FROM Q605A WITH ROW LABEL AND Q605B INITIALS IN PARENTHESES]

[LOOP Q420-Q440 THROUGH TWO PROVIDERS SELECTED AT Q405A. PIPED TEXT NEEDS TO BE ROW LABEL AND Q605B INITIALS IN PARENTHESES. IF NO PROVIDERS AVAILABLE TO SELECT AT Q410, SKIP TO Q473]

ALL QUALIFIED RESPONDENTS (S100r1)
Q420 Over the next several questions, we'd like you to think about [INSERT Q410], and how this healthcare provider has helped you manage your PCOS over time.

First, how frequently do you (or did you) typically have appointments with [INSERT Q410] **where your PCOS is discussed**?

1. Weekly
2. Monthly
3. Every other month
4. Every 3 months
5. Every 6 months
6. Yearly
96. Other, please specify: [INSERT TEXT BOX]

ALL QUALIFIED RESPONDENTS (S100r1)
Q426 Which of the following topics has [INSERT Q410] **ever** discussed with you?

Please select all that apply.

[RANDOMIZE; MULTIPLE SELECT]

1. Causes of PCOS
2. Treatments for PCOS (e.g., lifestyle modifications, prescription medications)
3. How PCOS is related to, or impacts, my other health conditions
4. Progression of PCOS (what to expect in the future)
96. Other, please specify: [INSERT TEXT BOX. ANCHO]
97. I do not recall [EXCLUSIVE. ANCHOR]

ALL QUALIFIED RESPONDENTS (S100r1)
Q430 Which resources has [INSERT Q410] **ever** provided to you to help with your understanding or management of PCOS?

Please select all that apply.

[MULTIPLE SELECT]

1. Reading material (i.e., pamphlet/brochure, book, magazine) about the condition
2. Website
3. Information on local support group
4. Smartphone app
96. Other, please specify: [INSERT TEXT BOX][ANCHOR]
97. My healthcare provider has not provided me with any resources [EXCLUSIVE]
98. I do not recall [EXCLUSIVE. ANCHOR]

ALL QUALIFIED RESPONDENTS (S100r1)
Q436 About how frequently (if at all) does [INSERT Q410] discuss your weight or weight management with you?

1. At every appointment
2. At most appointments
3. At some appointments
4. Rarely / at very few appointments
5. Never

HAS DISCUSSED WEIGHT (Q436r1-4)
Q440 When [INSERT Q410] discusses weight or weight management with you **during ongoing treatment and management**, which of the following have they discussed?

Please select all that apply.

[MULTIPLE SELECT. RANDOMIZE. GROUP CODES 1/2]

1. Explain the effect my weight has on my PCOS
2. Explain the effect my PCOS has on my weight
3. Help me set goals to improve my weight
4. Help me understand why I have excess weight
5. Make me aware of medications that will help me lose weight
6. Tell me about ways to lose weight (i.e., programs [e.g., Jenny Craig], diet, exercise)
7. Tell me about weight loss clinics
8. Suggest I see a registered Dietitian or nutritionist
96. Other, please specify: [INSERT TEXT BOX. ANCHOR]
97. I do not recall [ANCHOR. EXCLUSIVE]

[LOOP THROUGH ALL Q410 REMAINING HCPS]

[END LOOP]

ALL QUALIFIED RESPONDENTS (S100r1)
Q473 Which type of healthcare provider do you consider the "**coordinator" of your PCOS care**?

By "coordinator" please consider the healthcare provider who is primarily responsible for managing your PCOS in conjunction with any other conditions you have.

1. [INSERT Q605B PROVIDER LIST WITH INITIALS]
96. Other, please specify: [INSERT TEXT BOX]
97. I don't consider any healthcare providers to be the coordinator of my care [EXCLUSIVE]

ALL QUALIFIED RESPONDENTS (S100r1)
Q476 What are the biggest challenges you have in managing your PCOS?

Please select up to 3 challenges.

[MULTISELECT SELECT 3; RANDOMIZE]
1. Ensuring all health care providers have current/accurate information on my condition/treatments
2. Traveling significant distances to see specialists
3. Limited choice of specialists due to insurance coverage
4. Long wait times to see specialists
5. Limited ability to stay active and exercise
6. Affording doctor's appointments and treatments
7. Lack of education on PCOS cause and progression
8. Lack of alternative treatment options for PCOS
9. Using multiple medications to treat different PCOS symptoms
10. Lack of understanding and empathy from HCPs, family and/or friends
11. Difficulty achieving or sustaining weight loss over time
96. Other, please specify: [INSERT TEXT BOX, ANCHOR]
97. I don't have any challenges in managing my PCOS [EXCLUSIVE. ANCHOR]

ALL QUALIFIED RESPONDENTS (S100r1)
QC3 For quality control purposes, please select "5" from the list of options below.

1. 1
2. 2
3. 3
4. 4
5. 5

SECTION 500: IMPACT OF PCOS AND OBESITY

ALL QUALIFIED RESPONDENTS (S100r1)
Q500 Now we would like to understand how PCOS impacts your life. How much would you say your PCOS (and any associated symptoms or requirements for managing it) impacts each of the following aspects of your daily life?

Please use a scale from 1 to 7 where "1" means it "Doesn't impact at all" and "7" means "Greatly impacts."

[RANDOMIZE ROWS]	1 – Doesn't impact at all	2	3	4	5	6	7 – Greatly impacts
1. Your social relationships and activities							
2. Your family relationships and activities							
3. Your finances							
4. Doing household chores (cooking, cleaning, etc.)							
5. Running errands (go to the grocery store, doctors' appointments, etc.)							
6. Your job or career							
7. Being active / exercising							
8. Your hobbies							
9. Your mental / emotional health							

ALL QUALIFIED RESPONDENTS (S100r1)
Q510 To what extent do you feel your weight has impacted…

Please use the below scale where "1" means "Doesn't impact at all", and "7" means "Greatly impacts."

[RANDOMIZE ROWS]	1 – Doesn't impact at all	2	3	4	5	6	7 – Greatly impacts
1. Initial development of PCOS							
2. How quickly your PCOS progresses							
3. The severity of your PCOS symptoms							

ALL QUALIFIED RESPONDENTS (S100r1)
Q511 Which of the following statements do you believe is the <u>most accurate?</u>

Please consider cardiometabolic conditions to be conditions such as type 2 diabetes, heart disease, NASH, etc.

1. PCOS puts women at risk of cardiometabolic conditions
2. Cardiometabolic conditions put women at risk for PCOS
3. Both of the above are accurate
4. None of the above are accurate

ALL QUALIFIED RESPONDENTS (S100r1)
Q511A Which of the following statements do you believe is the most accurate?

1. PCOS causes obesity/excess weight
2. Obesity/excess weight causes PCOS
3. Both of the above are accurate
4. None of the above are accurate

ALL QUALIFIED RESPONDENTS (S100r1)
Q561 Please indicate how much you agree with each of the following…

Use a scale where "1" means "Do not agree at all" and "7" means "Completely agree."

1 - Do not agree at all 2 3 4 5 6 7 - Completely agree

[RANDOMIZE, CAROUSEL]

1. I could lose weight if I really set my mind to it.
2. I have a responsibility to actively contribute to my weight loss effort.
3. For me to lose weight, I would need to completely change my lifestyle.
4. I am past the point where I can lose weight on my own.
5. I am motivated to lose weight.
6. My weight is less important than my other conditions.
7. I do not feel comfortable bringing up my weight to my doctor unless they mention it first.
8. I know how to keep the weight off.

ALL QUALIFIED RESPONDENTS (S100r1)
Q516 In which of the following ways have you ever tried to lose weight (whether or not you were successful)?

Please select all that apply.

[RANDOMIZE. MULTIPLE SELECT}

1. General improvement in eating habits / reducing calories
2. Specific diet or diet program (Jenny Craig, Medifast, Weight Watchers, Elimination diets (avoiding fats, sugary beverages, carbohydrates, etc.)
3. Generally be more active / increase physical activity
4. A formal exercise program / Gym membership / Personal trainer
5. Over-the-counter (non-prescription) weight loss medication (dexatrim, vitamins, supplements, etc.)
6. Prescription weight loss medication
7. Visiting a nutritionist / dietitian or other weight loss specialist (may be in a weight loss clinic)
8. Weight loss surgery

96. Other, please specify: [MANDATORY TEXT BOX][ANCHOR]
97. I have never made a weight loss effort [EXCLUSIVE][ANCHOR]

ALL QUALIFIED RESPONDENTS (S100r1)
Q512B What prescription weight loss medications (if any) are you aware of?

[MANDATORY OPEN TEXT BOX]

USED RX WL MEDICATION (Q516r6)
Q517 How long after you were diagnosed with PCOS did you use a prescription weight loss medication?

If less than one year, enter 0 for "years" and then only enter in the number of months. If less than one month, enter "0" for both "months" and "years."

1. |_|_| years [RANGE: 0-S3]
2. |_|_| months [RANGE: 0-11]
3. I only used a prescription weight loss medication before my diagnosis with PCOS [EXCLUSIVE][ANCHOR]

ALL QUALIFIED RESPONDENTS (S100r1)
Q517B BTS MONTHS CALCULATION

[INSERT MONTHS: Q518Br1/2 + Q518Br2]

USED RX WL MEDICATION (Q516r6)
Q518A Which of the following weight loss medications have you used?

Please select all that apply.

[MULTISELECT. RANDOMIZE]

1. Liraglutide (Saxenda)
2. Orlistat (Xenical, Alli)
3. Naltrexone-bupropion (Contrave)
4. Phentermine (Adipex, Suprenza)
5. Phentermine/Topiramate ER (Qsymia)
6. Other, please specify: [MANDATORY TEXT BOX. ANCHOR]
7. I am not sure

USED RX WL MEDICATION (Q516R6)
Q518B Are you still using a prescription weight loss medication?

1. Yes
2. No

DISCONTINUED RX WL MEDICATION USE (Q518Br2)
Q518C For what reasons did you stop using a prescription weight loss medication?

[MANDATORY OPEN TEXT BOX]

ALL QUALIFIED RESPONDENTS (S100r1)
Q512A Please indicate how much you agree with the following regarding prescription medications for weight loss…

Use a scale where "1" means "Do not agree at all" and "7" means "Completely agree."

[RANDOMIZE, CAROUSEL]

1 - Do not agree at all 2 3 4 5 6 7- Completely agree

1. Prescription weight loss medications are more effective than other treatment options for weight loss.
2. I am likely to use new prescription weight loss medications in the future.
3. I am more likely to take a prescription medication than have a surgery (bariatric) to lose weight.
4. I am concerned about the side effects associated with prescription weight loss medications.
5. I would like my doctor to offer prescription weight loss medication to help me with my weight loss efforts.
6. Cost is a major barrier for me to consider prescription weight loss medications.
7. I would rather lose weight on my own than depend on medication.
8. I am concerned about the long-term safety associated with prescription weight loss medications.
9. I don't know enough about prescription weight loss medications to feel comfortable using them.

ALL QUALIFIED RESPONDENTS (S100r1)
Q514A Do you think it is necessary for a woman with PCOS who has excess weight or obesity to lose weight in order for PCOS symptoms (e.g., irregular periods, acne) to improve?

1. Yes
2. No
3. Not sure

ALL QUALIFIED RESPONDENTS (S100r1)
Q514B Do you think it is necessary for a woman with PCOS who has excess weight or obesity to lose weight in order to improve or restore fertility (e.g., achieve a successful pregnancy)?

1. Yes
2. No
3. Not sure

THINK WEIGHT LOSS IS NECESSARY FOR PREGNANCY (Q514Br1)
Q514C What percentage of body weight do you think a woman with PCOS and excess weight or obesity would need to lose in order to improve or restore fertility (e.g., achieve a successful pregnancy)?

[RANGE: 0-100]
|_|_|_| % of body weight necessary to lose

HAS SEEN MULTIPLE PROVIDERS WHO DISCUSSED WL (>1 PROVIDER SELECTED FROM Q615c6]
Q526 Now that we've learned about the healthcare providers you have seen, thinking across those who have discussed weight or weight loss with you, which do you feel has been the most helpful in supporting you in managing your weight?

Please select only one.

1. [SHOW ONLY THOSE SELECTED AT Q615c6]

97. None of these providers have been helpful

PROVIDER HAS BEEN HELPFUL (Q526NE97)
Q530 For what reasons do you feel [INSERT Q526] is the most helpful in supporting you in managing your weight? What do they do that is helpful?

Please be as specific as possible.

[LARGE MANDATORY TEXT BOX]

ALL QUALIFIED RESPONDENTS (S100r1)
Q535 For each of the following provider types, please indicate how comfortable you feel with each provider type addressing your **health holistically** (e.g., discussing and treating your PCOS, weight, and other conditions).

Please use a scale from 1 to 7 where "1" means "Not at all comfortable" and "7" means "Extremely comfortable."

Not at all comfortable						Extremely comfortable
1	2	3	4	5	6	7

1. Primary Care
2. OB/GYN
3. Bariatric Surgery
4. Obesity Medicine
5. General Endocrinology
6. Reproductive Endocrinology
7. Nutrition (i.e., Registered Dietitian/Nutritionist)

ALL QUALIFIED RESPONDENTS (S100r1)
Q540 Which, if any, of the following technologies have you used related to managing your PCOS? In managing your weight?

Please select all that apply.

[COLUMNS]
1. PCOS
2. Weight Management

[RANDOMIZE. MULTIPLE SELECT]
1 Online patient portal (to schedule appointments, view medical record, etc.)
2 Food / meal / nutrient tracking app or device
3 Exercise / activity tracking app or device
4 Other technology not listed here
5 I have not used any technology related to managing my health [EXCLUSIVE. ANCHOR]

[IF r4 SELECTED, POP UP ON NEXT PAGE ASKING "What other technology have you used for PCOS management" if r4c1 and "What other technology have you used for weight management?" if r4c2]

ALL QUALIFIED RESPONDENTS (S100r1)
Q543 Which of the following have you used as a source of information related to PCOS and which have you used as a source of information related to weight or weight management?

Please select all that apply.
[COLUMNS]
1. PCOS sources
2. Weight/Weight Management sources

[ROWS. RANDOM, MULTI-SELECT]

1. The Internet (Google, Social media, Websites)
2. Family and friends
3. Information from a healthcare provider
4. Wellness coach or personal trainer
5. Weight loss programs
6. Registered Dietitian or nutritionist (non-physician)
7. Peer support group
8. Smartphone apps
9. None of the above [ANCHOR, EXCLUSIVE]

USES INTERNET (Q543r1 ANY c1 AND/OR c2)
Q544 What specific websites do you consult for information on PCOS and/or weight management?

[MANDATORY TEXT BOX]

ALL QUALIFIED RESPONDENTS (S100r1)
Q545 How would you most prefer to learn about PCOS and weight management, whether you have used this source in the past or not?

*Please select your **top three** sources.*

[MULTIPLE SELECT. MAX 3 TO SELECT]

1. The Internet (Google, Social media, Websites)
2. Family and friends
3. Information from a healthcare provider
4. Wellness coach or personal trainer
6. Weight loss programs
7. Registered Dietitian or nutritionist (non-physician)
8. Peer support group
9. Smartphone apps
11. Other

RANKED OTHER SOURCE (Q545r11 RANKED)
Q545a What is the other source you would prefer to use to learn about PCOS and/or weight management?

[MANDATORY TEXT BOX]

ALL QUALIFIED RESPONDENTS (S100r1)
Q553 What are the **top 5 types of support that would be most helpful for you personally** to be successful with managing your weight?

Please select your top 5.

[SELECT 5 ONLY]

[RANDOMIZE]

1. Resources for family and friends to help understand how to be supportive
2. Specific meal plans to follow for weight management
3. Support groups (online or in-person) for those trying to lose weight
4. Programs offered at workplaces to help people lose weight
5. Financial support for healthy choices (gym membership, healthy foods)
6. App with weight loss tracking and ideas for healthy eating and physical activity
7. Weekly follow-up visits with a healthcare provider or Dietitian/nutritionist
8. Access to a physician who specializes in obesity

96. Other, please specify: [INSERT TEXT BOX. ANCHOR]
97. I don't need any type of support [EXCLUSIVE, ANCHOR]

SECTION 700: CLINICAL TRIALS

ALL QUALIFIED RESPONDENTS (S100r1)
Q700 How interested would you be in using a pharmaceutical treatment to assist in each of the following?

Use a scale where a "1" indicates "Not at all interested," and a "7" indicates "Extremely interested."

[COLUMNS]

Not at all interested						Extremely interested
1	2	3	4	5	6	7

[ROWS]
1. Fertility
2. Weight loss
3. Excess hair growth
4. Irregular/painful periods
5. All of the above (fertility, weight loss, irregular periods, hair growth)

ALL QUALIFIED RESPONDENTS (S100r1)
Q705 What, if anything, would you need to know to feel comfortable using a pharmaceutical treatment to assist in improving PCOS symptoms?

Please select all that apply.

[MANDATORY TEXT BOX[

ALL QUALIFIED RESPONDENTS (S100r1)
Q710 How interested would you be in participating in a clinical trial investigating the use of a pharmaceutical treatment to assist in weight loss and improvement in PCOS symptoms?

Use a scale where a "1" indicates "Not at all interested," and a "7" indicates "Extremely interested."

Not at all interested						Extremely interested
1	2	3	4	5	6	7

SECTION 100: DEMOGRAPHICS

ALL QUALIFIED RESPONDENTS (S100r1)
Q103 What is the highest degree you received?

1. High school diploma or the equivalent (GED)
2. Associate degree
3. Bachelor's degree
4. Master's degree
5. Professional degree (MD, DDS, DVM, LLB, JD, DD)
6. Doctorate degree (Ph.D. or Ed.D.)

97. None of the above

ALL QUALIFIED RESPONDENTS (S100r1)
Q105 What best describes your employment status?

1. Employed full-time
2. Employed part-time
3. A homemaker
4. A full-time student
5. Retired
6. Unable to work for health reasons
7. Unemployed
8. Other

ALL QUALIFIED RESPONDENTS (S100r1)
Q110 In 2019, what was your household's total yearly income <u>before</u> taxes?

Please remember that your individual information will never be shared. These questions are only used to ensure a representative mix of respondents is achieved.

1. Under $25,000
2. $25,000 - $49,999
3. $50,000 - $74,999
4. $75,000 - $99,999
5. $100,000 - $149,999
6. $150,000 or more
7. Decline to answer

ALL QUALIFIED RESPONDENTS (S100r1)
Q115 Are you currently covered by any of the following types of health insurance or health coverage plans?

Please mark "yes" or "no" for each type of coverage.

[COLUMNS]
1. Yes
2. No

[ROWS]

1. Insurance through a current or former employer or union
2. Insurance purchased directly from an insurance company
3. Medicare, for people 65 and older, or people with certain disabilities
4. Medicaid, Medical Assistance, or any kind of government-assistance plan for those with low incomes or a disability
5. TRICARE or other military healthcare
6. VA (including those who have ever used or enrolled for VA health care)
7. Indian Health Service
8. Any other type of health insurance or health coverage plan

[IF HAS HEALTH INSURANCE (Q115r1-8c1), ASK Q165. ALL OTHERS JUMP TO NEXT QUESTION.]

HAS HEALTH INSURANCE (Q115r1-8c1)
Q165 Do you currently have a health insurance plan that helps pay for the cost of prescription drugs?

1. Yes
2. No
3. Not sure
