# Supplementary material for: Polycystic Ovary Syndrome and Obesity: A Cross-Sectional Survey of Patients and Obstetricians/Gynecologists
Source: J Womens Health (Larchmt). 2023 Jun 6;32(6):723–31. doi: 10.1089/jwh.2022.0471 (PMC10278022; doi:10.1089/jwh.2022.0471)
Supplement: Supplemental data [file Supp_AppS2.pdf]

Appendix Survey A2. Healthcare professional survey

PCOS Medical Interface Mapping Research – Questionnaire – HCPs
October 7, 2020

Methodology

25-minute online survey

Screening Criteria:

- Consents to terms
- Agrees to AE reporting
- Physician
- Qualified specialty – PCP, OB/GYN, General Endos, Reproductive Endo
- Board certified in specialty
- Works in the US but not ME or VT
- In practice 3-25 years
- Does not work in a government/VA hospital or ASC
- Treats a minimum number of patients with PCOS and obesity in the past month (5 if PCP or General Endo; 10 if OB/GYN or Reproductive Endo)

SECTION S: SCREENER

ALL RESPONDENTS
S0 Thank you for your interest in this survey. We appreciate your willingness to participate in this important research on healthcare issues. Before participating, KJT Group requires you to review the following information:

- KJT Group is a **global market research company**.
- Your responses to this survey will **help the sponsor design new products/services to meet patient needs**.
- Your responses will be kept **strictly confidential** and will never be associated with your name (double-blind).
- We expect, on average, it will take respondents like yourself **25 minutes** to participate.
- Your **participation is voluntary**, and you may choose to stop participating at any time (withdraw consent).

Do you consent to these terms and wish to continue with the survey?

1. Yes **CONTINUE**
2. No **TERMINATE**

[IF CONSENTS TO TERMS (S0r1) ASK AE1. ELSE TERMINATE]

CONSENTS TO TERMS (S0r1)
AE1 We are required to pass on to the pharmaceutical company sponsoring the study details of adverse events and/or other safety information - hereinafter referred to as safety information - that are mentioned during this study. Although what you say will be treated in confidence, should you mention safety information during the study, we will need to report it even if you have already reported it to the company or regulatory authorities.

In relation to reporting safety information, situation we need to know if you are willing to waive the confidentiality given to you under the Market Research Codes of Conduct. In the event that you waive confidentiality in relation to safety information reporting, any personal data provided during the reporting will be processed as follows:

a) Any personal data in relation to the safety information reported will be forwarded to the project sponsor; and

b) The project sponsor will record any safety information, including personal data received in the sponsor's global database, in the interests of patient safety and in compliance with all applicable global laws and regulations; and

c) During the reporting of safety information, the project sponsor will not disclose such personal data to any un-associated third parties, with the exception of any disclosures required by applicable law, regulation or the order of a competent authority.

Do you agree to waive the confidentiality given to you under the Market Research Codes of Conduct in relation to any safety information you report to us? If you agree, your contact details will be forwarded to the sponsor's Safety department for the express and sole purpose of follow-up of such report(s). Details of safety information maybe reported to regulatory authorities along with your personal data. All other information provided by you in this study will remain confidential. If you prefer to preserve the confidentiality of this information, please select 'I do not agree'. If you do so, you can still participate in this survey.

1. I agree   Please enter your email here: [MANDATORY OPEN TEXT BOX. MUST BE IN EMAIL FORMAT BUT NO VALIDATION REQUIRED]
2. I do not agree

[IF AGREES (AE1r1) ASK AE2. IF DISAGREES (AE1r2) JUMP TO AE3]

AGREES (AE1r1)
AE2 Thank you. Please note that if your email address is provided during the Adverse Event, other safety information or product complaints reporting, this will not be linked in any way to your responses.

Are you happy to proceed with this research?

1. Yes **CONTINUE**
2. No **TERMINATE**

[IF YES (AE2r1) JUMP TO S3. ELSE TERMINATE]

DOES NOT AGREE (AE1r2)
AE3 If we become aware of safety information we are obliged to report this to the pharmaceutical company. We will file this report without giving any of your details.

Are you happy to proceed with this research?

1. Yes **CONTINUE**
2. No **TERMINATE**

[IF HAPPY TO PROCEED (AE3r1) ASK S3. ELSE TERMINATE]

AGREES TO AE REPORTING (AE2r1 OR AE3r1)
S3 To begin, we would like to gather some basic information to be used for categorization purposes.

Which of the following best describes your professional title?

[ALPHA SORT]
1. Physician **CONTINUE**
2. Nurse Practitioner **TERMINATE**
3. Physician Assistant **TERMINATE**
4. Physical Therapist **TERMINATE**
5. Nurse **TERMINATE**
6. Medical Assistant **TERMINATE**
7. Other, please specify [MANDATORY TEXT BOX] [ANCHOR] **TERMINATE**

[IF PHYSICIAN (S3r1) ASK S5; ELSE TERMINATE]

PHYSICIAN (S3r1)
S5 How many years have you been in practice beyond your residency or fellowship?

If you are still in your residency, are currently a fellow, or have not been in practice for at least one year, please enter "0" (zero).

[RANGE: 0-50]
|_|_|_| years **CONTINUE IF 3-30; ALL ELSE CONTINUE BUT MARK AS NOT QUALIFIED**

PHYSICIAN (S3r1)
S10 What is your primary specialty?

[ALPHA SORT]
1. Obstetrics/Gynecology **CONTINUE**
2. Endocrinology - Reproductive **CONTINUE**
3. Endocrinology - General **CONTINUE**
4. Family Practice **CONTINUE**
5. General Practice **CONTINUE**
6. Internal Medicine **CONTINUE**
7. Bariatrics/Obesity Medicine **TERMINATE**
8. General Surgery **TERMINATE**
9. Maternal Fetal Medicine **TERMINATE**
10. Rheumatology **TERMINATE**
11. Orthopedic **TERMINATE**
12. Pulmonology **TERMINATE**
13. Neurology **TERMINATE**
14. Nephrology **TERMINATE**
15. Gastroenterology **TERMINATE**
16. Cardiology **TERMINATE**
96. Other, please specify: [MANDATORY TEXT BOX. ANCHOR] **CONTINUE BUT MARK AS NOT QUALIFIED**

[IF OB/GYN (S10r1) ASK S10B; IF OTHER PHYSICIAN IN QUALIFIED OR POTENTIALLY QUALIFIED SPECIALTY (10r2-6,96) JUMP TO S10A; ELSE TERMINATE]

QUALIFIED OB/GYN (S10r1)
S10B Do you specialize in reproductive endocrinology?

1. Yes
2. No

PHYSICIAN IN QUALIFIED OR POTENTIALLY QUALIFIED SPECIALTY (S10r1-6,96)
S10A Are you board certified or board eligible in your primary specialty?

1. Yes **CONTINUE**
2. No **CONTINUE BUT MARK AS NOT QUALIFIED**

PCP WHO IS BOARD CERTIFIED ((S10r4-6 AND S10Ar1)
S12 Regardless of your official medical specialization (or the specialty of your office), does your current practice and caseload focus primarily on the treatment of obesity or excess weight?

1. Yes
2. No

PHYSICIAN IN QUALIFIED OR POTENTIALLY QUALIFIED SPECIALTY (S3r1 AND S10r1-6,96)
S15 In what state is the facility where you primarily work?

If you work in more than one state, please select the state where you work the majority of the time.

[INSERT STATE DROP DOWN] **CONTINUE IF US AND NOT ME OR VT**

[CONTINUE IF WORKS IN US AND NOT VT OR ME (S15r1=US AND NOT ME OR VT); ELSE TERMINATE]

WORKS IN US AND NOT VT OR ME (S15r1=US AND NOT ME OR VT)
S20 HIDDEN QUESTION FOR REGION

1. Northeast
   [S15=CT, MA, NH, NJ, NY, PA, RI, VT]
2. Midwest
   [S15=IA, IL, IN, KS, MI, MN, MO, ND, NE, OH, SD, WI]
3. South
   [S15=AL, AR, DC, DE, FL, GA, KY, LA, MD, MS, NC, OK, SC, TN, TX, VA, WV]
4. West
   [S15=AK, AZ, CA, CO, HI, ID, MT, NM, NV, OR, UT, WA, WY]
5. Outside of US
   [ALL OTHERS]

WORKS IN US AND NOT VT OR ME (S15r1=US AND NOT ME OR VT)
S25 Which of the following best describes the facility where you primarily see patients?

[ALPHA SORT]

1. Academic hospital (teaching hospital/university)	**CONTINUE**
2. Community hospital	**CONTINUE**
3. Private solo practice	**CONTINUE**
4. Hospital affiliated practice	**CONTINUE**
5. Private single-specialty group practice	**CONTINUE**
6. Private multi-specialty group practice	**CONTINUE**
7. Ambulatory surgery center	**TERMINATE**
8. Government or VA hospital	**TERMINATE**
9. Other, please specify [MANDATORY TEXT BOX] [ANCHOR]	**TERMINATE**

[IF NOT BASED IN A GOVT/VA HOSPITAL OR ASC OR OTHER FACILITY (S30r1-6) ASK S30; ELSE TERMINATE]

NOT BASED IN A GOVT/VA HOSPITAL OR ASC OR OTHER FACILITY (S25r1-6)
S30 In the past month, approximately how many total adult patients (age 18 and older) did you personally see/treat across all conditions and across all care settings (hospitals, outpatient clinics, etc.), including in-person and telehealth?

Your best estimate is fine.

[RANGE: 0-9999]
Patient(s) in the past month [_|_I_I_]

NOT BASED IN A GOVT/VA HOSPITAL OR ASC OR OTHER FACILITY (S25r1-6)
S31 In the past month, out of the [INSERT S30] adult patients you saw/treated, approximately how many have obesity? Please consider a person with obesity as someone with a **Body Mass Index (BMI) of 30 or greater** with or without comorbidities.

Please consider all persons that have obesity, whether or not their weight was discussed during their visit. Your best estimate is fine.

[RANGE: 0-S30]
Persons with obesity in the <u>past month</u> [_|_I_I_]

NOT BASED IN A GOVT/VA HOSPITAL OR ASC OR OTHER FACILITY (S25r1-6)

S35 Considering the [INSERT S31] adult patients you saw in the past month **<u>with obesity</u>**, approximately how many of these patients are **<u>also diagnosed with each of the following conditions</u>**?

Please consider a person with obesity as someone with a Body Mass Index (BMI) of 30 or greater with or without comorbidities. Your best estimate is fine.

[RANDOMIZE; GROUP CODES 2/4]	RANGE: 0-S31
1. PCOS (Polycystic ovary syndrome)	|_|_|_| **CONTINUE IF PCP OR GEN ENDO OR OTHER AND 5+, OR OB/GYN OR REPRODUCTIVE ENDO AND 10+; ALL ELSE CONTINUE BUT MARK AS NOT QUALIFIED**
2. Infertility	|_|_|_|
3. Hyperandrogenism	|_|_|_|
4. Anovulation or oligo-anovulation	|_|_|_|
5. Hirsutism	|_|_|_|
6. Pre-diabetes	|_|_|_|
7. Type 2 Diabetes	|_|_|_|

ALL RESPONDENTS

S100 OVERALL QUOTA

1. QUALIFIED PHYSICIAN **[N=305]**
- o AGREES TO AE REPORTING (AE2r1 OR AE3r1)
- O PHYSICIAN (S3R1)
- O QUALIFIED SPECIALTY (S10R1-6)
- O BOARD CERTIFIED IN QUALIFIED SPECIALTY (S10AR1)
- O PCP, OB/GYN OR GEN ENDO AND IN PRACTICE 3-25 YEARS (S10R3-6 OR S10R1 AND S10BR2 AND S5R1=3-25) OR REP ENDO AND IN PRACTICE 3-30 YEARS (S10R2 OR S10BR1 AND S5R1=3-30)
- O DOES NOT WORK IN A GOVERNMENT/VA HOSPITAL OR ASC (S25R1-6)
- O TREATS A MINIMUM NUMBER OF PWPCO (5 IF PCP OR GENERAL ENDO; 10 IF OB/GYN OR REPRODUCTIVE ENDO; S35R1=10+ IF S10R1-2 ELSE S35R1=5+)

99. NOT QUALIFIED **[N=999]**

ALL QUALIFIED RESPONDENTS (S100r1)

S105 SPECIALTY QUOTAS

1. OB/GYN (S10r1 AND S10Br2)	[N=125]
2. REPRODUCTIVE ENDO (S10r2 OR S10Br1)	[N=30]
3. GENERAL ENDO (S10r3)	[N=75]
4. PCP (S10r4-6)	[N=75]

ALL QUALIFIED PCPs (S105r4)

S110 SOFT QUOTA OBESITY SPECIALIST

1. Obesity Specialist	(S12r1)	N=999
2. Non-Obesity Specialist	(S12r2)	N=999

SECTION 200: DIAGNOSIS AND MANAGEMENT OF PWPCO

ALL QUALIFIED RESPONDENTS (S100r1)
Q200 You have qualified for the full survey. Thank you for your responses thus far. The remainder of this survey should take approximately 22 minutes to complete. As a reminder, your responses to this survey are critical to the success of this research in helping the sponsor design new products/services to help you support your patients' needs. Your responses will be kept strictly confidential and only reported in combination with other respondents' data. In addition, you may be asked certain questions for quality control purposes.

ALL QUALIFIED RESPONDENTS (S100r1)
Q205 For the remainder of the survey please focus on your patients who are **diagnosed with polycystic ovary syndrome (PCOS) and also have obesity (BMI of 30 or greater).**

You indicated you see [INSERT S35r1] adult patients per month who have PCOS and obesity. Approximately what proportion of these patients fall into each of the age ranges below?

Your best estimate will do. Your answers must sum to 100%.

[RANGE 0-100. SHOW CONSTANT SUM INDICATOR. FORCE SUM 100]

	Proportion of patients with PCOS and obesity
1. Age 18-25	|_|_|_|%
2. Age 26-40	|_|_|_|%
3. Age 41-55	|_|_|_|%
4. Over 55 years old	|_|_|_|%

ALL QUALIFIED RESPONDENTS (S100r1)
Q220 Still thinking of your patients who have PCOS and obesity, approximately what proportion of these patients are diagnosed with PCOS in the following ways?

Your best estimate will do. Your answers must sum to 100%.

[RANGE 0-100. SHOW CONSTANT SUM INDICATOR. FORCE SUM 100. DO NOT FORCE ZEROS]

	Proportion of patients with PCOS and obesity
1. I personally diagnose them with PCOS	|_|_|_|%
2. They are diagnosed by someone else	|_|_|_|%
3. I refer them to someone else for diagnosis	|_|_|_|%

[IF DIAGNOSES SOME PWPCO (Q220r1>0) ASK Q230. ELSE SKIP TO PN BEFORE Q232A]

DIAGNOSES SOME PWPCO (Q220r1>0)
Q230 Thinking of your patients with PCOS and obesity that **you personally diagnose with PCOS**, we'd like to understand how you first start the process of diagnosing these patients.

First please think about when you first hear about their PCOS symptoms before they are formally diagnosed. What proportion of the time are you hearing about symptoms in the following scenarios?

Your best estimate will do. Your answers must sum to 100%.

[RANGE 0-100. SHOW CONSTANT SUM INDICATOR. FORCE SUM 100]

	Proportion of patients with PCOS and obesity **<u>who you diagnose</u>**
1. Appointment made for a well-visit or annual exam	|_|_|_|%
2. Appointment made specifically to discuss PCOS symptoms	|_|_|_|%
3. Appointment made for another condition, but PCOS symptoms came up	|_|_|_|%
4. Other scenario not listed	|_|_|_|%

DIAGNOSES SOME PWPCO (Q220r1>0)
Q232 When a patient who has obesity (BMI greater than 30) comes to you with symptoms that may indicate PCOS, what do you typically do to confirm a diagnosis of PCOS?

Please select all that apply.

[RANDOMIZE; MULTIPLE SELECT]

1. PCOS-related blood testing (e.g., TSH, FSH, DHEAS, androgen)
2. Blood testing to rule out another comorbidity (e.g., A1C, thyroid)
3. Pelvic exam
4. Ultrasound
5. Review patient medical history and/or weight history
6. Physical presentation of symptoms (e.g., presence of acne, excess weight, body hair)
7. Refer to a specialist/another physician

96. Other, please specify [MANDATORY TEXT BOX. ANCHOR]

[IF REFERS TO OTHER PHYSICIAN FOR DIAGNOSIS (Q232/10 OR Q220r3>0) ASK S232A. ELSE SKIP TO Q235]

REFERS TO OTHER PHYSICIAN FOR DIAGNOSIS (Q232r7 OR Q220r3>0)
Q232A To what provider(s) do you typically refer patients to <u>confirm their diagnosis</u> of PCOS?

Please select all that apply.

[ALPHA SORT. MULTIPLE SELECT]

1. [IF PCP S105r4 "Another"] Primary Care Provider
2. [IF OB/GYN S105r1 "Another"] OB/GYN
3. [IF REI S105r2 "Another"] Reproductive Endocrinologist
4. [IF Gen Endo S105r3 "Another"] General Endocrinologist

96. Other, please specify: [MANDATORY TEXT BOX. ANCHOR]

DIAGNOSES SOME PWPCO (Q220r1>0)
Q230B Please indicate your level of comfort with making the following diagnoses among your patients with PCOS and obesity.

Use a scale where a "1" indicates "Not at all comfortable," and a "7" indicates "Extremely comfortable."

[GRID]

Not at all comfortable					Extremely comfortable	
1	2	3	4	5	6	7

1. Diagnosis of PCOS
2. Diagnosis of obesity

ALL QUALIFIED RESPONDENTS (S100r1)
Q235 When you diagnose a patient who has PCOS and obesity, or see them for the first time (after diagnosis), which of the following topics do you typically cover in that conversation?

Please select all that apply.

[MULTIPLE SELECT]
1. Cause of PCOS
2. Treatments for PCOS (e.g., lifestyle modifications, prescription medications)
3. How PCOS is related to or impacts other health conditions
4. Progression of PCOS
5. Referral to a specialist or other healthcare provider for PCOS
96. Other, please specify: [MANDATORY TEXT BOX]
97. I do not typically discuss their PCOS with them [EXCLUSIVE]

ALL QUALIFIED RESPONDENTS (S100r1)
Q238 When you diagnose a patient who has PCOS and obesity or see them for the first time (after diagnosis): what, if any, resources do you provide to you to help your patients' understanding or management of PCOS?

Please select all that apply.

[MULTIPLE SELECT. RANDOMIZE]
1. Reading material (i.e., pamphlet/brochure, book, magazine) about the condition
2. Website
3. Information on local support group
4. Smartphone app
96. Other, please specify: [MANDATORY TEXT BOX] [ANCHOR]
97. I do not provide any resources [EXCLUSIVE][ANCHOR]

DIAGNOSES SOME PWPCO (Q220_1>0)
Q239 How frequently do you typically have follow-up appointments <u>specific to PCOS</u> with your patients who have PCOS and obesity after diagnosis?

1. Weekly
2. Monthly
3. Bi-monthly (every other month)
4. Quarterly (every 3 months)
5. Bi-annually (every 6 months)
6. Yearly
96. Other, please specify: [MANDATORY TEXT BOX. ANCHOR]
97. I typically do not have follow-up appointments with patients specific to their PCOS [EXCLUSIVE][ANCHOR]

HAS FOLLOW UP APPOINTMENTS (Q239NE97)
Q239B Once a patient who has obesity is diagnosed with PCOS, who in your office typically handles <u>follow-up appointments</u> regarding their <u>PCOS treatment and management</u>?

1. I primarily conduct follow-up visits with these patients

2. Nurse Practitioner or Physician Assistant
96. Other, please specify: [MANDATORY TEXT BOX. ANCHOR]

ALL QUALIFIED RESPONDENTS (S100r1)
Q239A Thinking of your patients who have PCOS and obesity, approximately what proportion of these patients do you treat personally or refer for treatment of PCOS?

Your best estimate will do.

[RANGE 0-100. DO NOT FORCE SUM.]

	Proportion of patients with PCOS and obesity
1. I initiate PCOS treatment for patients	|_|_|_|%
2. I adjust PCOS treatment for patients	|_|_|_|%
3. I refer to someone else for treatment of PCOS	|_|_|_|%

TREATS SOME PWPCO (Q239Ar1>0 OR Q239Ar2>0)
Q240 Which, if any, clinical guidelines do you follow for the treatment and management of **PCOS**?

Please select all that apply.

[MULTIPLE SELECT. ALPHA SORT]
1. American College of Obstetricians and Gynecologists (ACOG)
2. American Association of Clinical Endocrinologists / American College of Endocrinology / Androgen Excess Society (AACE/ACE/AES)
3. National Health and Medical Research Council / European Society of Human Reproduction and Embryology / American Society for Reproductive Medicine (NHMRC/ESHRE/ASRM)
4. Endocrine Society
96. Other, please specify [MANDATORY TEXT BOX. ANCHOR]
97. None [EXCLUSIVE. ANCHOR]

TREATS SOME PWPCO (Q239Ar1>0 OR Q239Ar2>0)
Q243 What type of treatment(s) do you personally prescribe or recommend to patients with PCOS and obesity **for the treatment of PCOS symptoms**?

Please provide your response for both newly diagnosed patients, as well as those who are on ongoing treatment/management.

Please select all that apply.

[COLUMNS]
1. For patients newly diagnosed with PCOS
2. For ongoing PCOS treatment and management of PCOS

[ROWS. RANDOMIZE]
1. Oral contraceptives
2. Metformin (Glucophage, Riomet)
3. Letrozole (Femara) / Clomid (Clomiphene)
4. Spironolactone (CaroSpir, Aldactone)
5. Provera (Depo-Provera)
6. General improvements in lifestyle (e.g., improving eating habits, increasing physical activity)

7. Specific diet or diet program (Jenny Craig, Medifast, Weight Watchers, Elimination diets, etc.)
8. Prescription weight loss medication (e.g., Saxenda, Xenical, Qsymia, Contrave)
96. Other, please specify [MANDATORY TEXT BOX. ANCHOR]
97. I do not prescribe or recommend treatment [ANCHOR, EXCLUSIVE]

TREATS SOME PWPCO (Q239Ar1>0 OR Q239Ar2>0)
Q256 How do you determine when a change in treatment is needed for a patient with PCOS and obesity?

Please select all that apply.

[MULTIPLE SELECT. RANDOMIZE]
1. The patient is not getting adequate relief from PCOS symptoms on current treatment and/or is dissatisfied with results of treatment
2. Current treatment regimen is not well tolerated by the patient
3. Change in patient's insurance coverage limits access to current therapies
4. The patient specifically requests an alternative treatment that is appropriate for their condition
5. The patient is unwilling to continue the current treatment regimen
6. The patient's comorbidities or medications for comorbidities restrict treatment options
7. When severity of PCOS significantly increases (e.g., from moderate to severe)
8. Changes in patient bloodwork (e.g., hormone levels, comorbidity markers)
96. Other, please specify: [MANDATORY TEXT BOX. ANCHOR]
97. I do not typically determine this (someone else does) [ANCHOR; EXCLUSIVE]

REFERS SOME PATIENTS FOR TREATMENT (Q239Ar3>0)
Q260 What percent of your patients with PCOS and obesity do you typically refer to each type of healthcare provider listed below for ongoing treatment and management of PCOS?

Your responses do not need to add to 100%.

[ALPHA SORT. DO NOT FORCE ZEROS. RANGE 0-100]	Proportion of patients with PCOS and obesity
1. [IF PCP S105r4 "Another"] Primary Care Provider	|_|_|_|%
2. [IF OB/GYN S105r1 "Another"] OB/GYN	|_|_|_|%
3. [IF REI S105r2 "Another"] Reproductive Endocrinologist	|_|_|_|%
4. [IF Gen Endo S105r3 "Another"] General Endocrinologist	|_|_|_|%
5. Bariatric Surgeon	|_|_|_|%
6. Obesity Medicine Specialist	|_|_|_|%
7. Registered Dietitian/Nutritionist	|_|_|_|%
96. Other provider [ANCHOR]	|_|_|_|%
97. I do not typically refer any of my patients for ongoing management [EXCLUSIVE. ANCHOR]	

[IF REFER TO SPECIALISTS FOR TX/MANAGEMENT (S100/1-4 AND Q260 ANY>0) ASK Q261. IF REFER TO OTHER PROVIDER (Q260R96>1) ASK Q260A. ELSE SKIP TO Q265]

REFER TO OTHER PROVIDER (Q260r96>0)
Q260A To what other provider do you refer?

[MANDATORY TEXT BOX]

HCPS WHO REFER TO SPECIALISTS FOR TX/MANAGEMENT (S100/1-4 AND Q260 ANY>0)
Q261 For each specialist that you refer your patients with PCOS and obesity to for ongoing treatment and management of PCOS, what are the primary reason(s) for doing so?

I primarily refer to this type of provider because the patient…

[COLUMNS; SHOW ONLY IF RESPONSE AT Q260 >0] [ONE RESPONSE PER COLUMN]

1. PCP
2. OB/GYN
3. Reproductive Endo
4. General Endo
5. Bariatric Surgeon
6. Obesity Medicine
7. Dietitian/Nutritionist
8. [INSERT Q260A IF Q260r96>1]

[ROWS, MULTISELECT.]

1. [DO NOT USE]
2. Additional management/treatment of PCOS
3. Needs to lose weight (i.e., to undergo surgery, to improve PCOS prognosis and progression)
4. Struggles with lifestyle modifications
5. Needs treatment for comorbidities (e.g., type 2 diabetes, NASH, cardiovascular disease, chronic kidney disease)
6. Needs treatment for other PCOS symptoms outside of my specialty
7. The patient's PCOS symptoms have worsened
8. Seeking pregnancy
9. Requires medications not offered in my practice
96. Other reason [POP UP TEXT BOX ON NEXT PAGE IF SELECTED]

REFERS TO BARIATRIC SURGEONS (Q260r5 > 0)

Q263 How would you describe your level of collaboration with the **bariatric surgeon(s) you refer to** when managing a patient with PCOS and obesity?

Use a scale where a "0" indicates "Do not collaborate at all," and a "10" indicates "Actively collaborate."

Do not collaborate at all									Actively collaborate	
0	1	2	3	4	5	6	7	8	9	10

ALL QUALIFIED RESPONDENTS (S100r1)

Q265 What providers **refer patients <u>to you</u>** for <u>ongoing treatment and management of PCOS</u>?

Please select all that apply.

[ALPHA SORT. DO NOT FORCE ZEROS]

1. [IF PCP S105r4 "Another"] Primary Care Provider
2. [IF OB/GYN S105r1 "Another"] OB/GYN
3. [IF REI S105r2 "Another"] Reproductive Endocrinologist
4. [IF Gen Endo S105r3 "Another"] General Endocrinologist
5. Bariatric Surgeon
6. Obesity Medicine Specialist
7. Registered Dietitian/Nutritionist
96. Other, please specify: [MANDATORY TEXT BOX. ANCHOR]
97. N/A - other providers do not typically refer to me for this reason [EXCLUSIVE. ANCHOR]

ALL QUALIFIED RESPONDENTS (S100r1)
Q268 What type of healthcare provider do you typically think of as the "coordinator of care" of patients who have PCOS and obesity?

By "coordinator" please consider the healthcare provider who is primarily responsible for managing the patient's PCOS in conjunction with any other comorbidities they have.

[ROWS, ALPHA SORT.]

1. I am the coordinator of care [ANCHOR AT TOP]
2. [IF PCP S105r4 "Another"] Primary Care Provider
3. [IF OB/GYN S105r1 "Another"] OB/GYN
4. [IF REI S105r2 "Another"] Reproductive Endocrinologist
5. [IF Gen Endo S105r3 "Another"] General Endocrinologist
6. Bariatric Surgeon
7. Obesity Medicine Specialist
8. Registered Dietitian/Nutritionist
96. Other, please specify [INSERT TEXT BOX. ANCHOR]

TREATS SOME PWPCO (Q239Ar1>0 OR Q239Ar2>0)
Q273 In your opinion, what are the **top three** reasons patients who have PCOS and obesity **stop seeing you for management of their PCOS**?

Please select up to three responses.

[RANDOMIZE. MULTIPLE SELECT. ONLY ALLOW THREE SELECTIONS]

1. Personal financial limitations
2. Change in insurance coverage
3. Lack of treatment efficacy
4. Unwilling to comply with treatment algorithm
5. Transportation difficulties
6. Expense of multiple visits
7. Improvement in symptoms
8. Using self-care/OTC measures
9. Difficulty with weight loss/management
10. I do not ask/require them to schedule a follow-up
96. Other, please specify [INSERT TEXT BOX. ANCHOR]

ALL QUALIFIED RESPONDENTS (S100r1)
Q276 To what extent to do you feel a patient's obesity impacts…

Please use the below scale where "1" means "Doesn't impact at all", and 7 means "Greatly impacts."

[RANDOMIZE ROWS]	1 – Doesn't impact at all	2	3	4	5	6	7 – Greatly impacts
1. Initial development of PCOS							
2. How quickly PCOS progresses							
3. Severity of PCOS symptoms							

ALL QUALIFIED RESPONDENTS (S100r1)
Q280 Which of the following statements do you believe is the most accurate?

Please consider cardiometabolic conditions to be conditions such as type 2 diabetes, cardiovascular disease, NASH, etc.

1. PCOS puts women at risk of cardiometabolic conditions
2. Cardiometabolic conditions put women at risk for PCOS
3. Both of the above are accurate
4. None of the above are accurate

ALL QUALIFIED RESPONDENTS (S100r1)
Q280A Which of the following statements do you believe is the most accurate?

1. PCOS causes obesity/excess weight
2. Obesity/excess weight causes PCOS
3. Both of the above are accurate
4. None of the above are accurate

ALL QUALIFIED RESPONDENTS (S100r1)
Q280B What is your level of interest in addressing the following among your patients with PCOS and obesity?

Use a scale where a "1" indicates "Not at all interested," and a "7" indicates "Extremely interested."

[COLUMNS]

Not at all interested					Extremely interested	
1	2	3	4	5	6	7

[ROWS]

1. Cardiometabolic comorbidities
2. Obesity/excess weight

SECTION 300: OBESITY MANAGEMENT

ALL QUALIFIED RESPONDENTS (S100r1)
Q300 Have you received any advanced formal training in the treatment of obesity/weight management beyond medical school training?

1. Yes
2. No

RECEIVED TRAINING (Q300r1)
Q300A What advanced formal training have you received for the treatment of obesity/weight management beyond medical school training?

[MANDATORY LARGE OPEN TEXT BOX]

ALL QUALIFIED RESPONDENTS (S100r1)
Q305 To what extent do you agree with the following?

Use a scale where a "1" indicates "Strongly disagree," and a "7" indicates "Strongly agree."

Strongly disagree					Strongly agree	
1	2	3	4	5	6	7

1. I am **confident** in handling obesity/weight management with my PCOS patients
2. I am **fully informed** to handle obesity/weight management with my PCOS patients
3. I am **interested** in handling obesity/weight management with my PCOS patients

ALL QUALIFIED RESPONDENTS (S100r1)
Q306 Would you be interested in receiving additional training or support on how to handle obesity/weight management, specifically for your patients with PCOS and obesity?

1. Yes
2. No

INTERESTED IN ADDITIONAL TRAINING (Q306r1)
Q306A What kind of additional training or support on obesity/weight management would you be interested in?

[MANDATORY LARGE TEXT BOX]

ALL QUALIFIED RESPONDENTS (S100r1)
Q307A What is your level of interest in learning (or learning more) about obesity and weight management, specifically for your patients with PCOS and obesity?

Use a scale where a "1" indicates "Not at all interested," and a "7" indicates "Extremely interested."

Not at all interested					Extremely interested	
1	2	3	4	5	6	7

ALL QUALIFIED RESPONDENTS (S100r1)
Q308 What level of influence would information and guidance from thought leaders in each of the following specialty areas have on your motivation to counsel patients with PCOS and obesity on weight management?

Use a scale where a "1" indicates "Not at all influential," and a "7" indicates "Extremely influential."

Not at all influential					Extremely influential	
1	2	3	4	5	6	7

[RANDOMIZE]

1. Obesity Medicine
2. Bariatric Surgery
3. Primary Care
4. General Endocrinology

5. Reproductive Endocrinology
6. OB/GYN
7. Nutrition

ALL QUALIFIED RESPONDENTS (S100r1)
Q307 We would like to understand your preferred sources for information about obesity and weight management. Please rank your **top five** sources for learning about weight management in the future.

Please click or drag and drop to rank, starting with '1' meaning "most preferred," '2' meaning "second most preferred," and so on until your top five are ranked.

[RANK TOP 5; DRAG AND DROP. RANDOMIZE]
1. Press articles from medical press
2. Scientific journals
3. Paper brochures
4. Professional organization websites
5. Congresses/Conferences
6. In-person courses/workshops
7. Discussions with peers/colleagues
8. Online courses/webinars
9. E-books
10. Podcasts
11. Patient testimonials
12. Case studies
13. Social media
14. General online searches
15. Discussions with pharmaceutical sales representatives
16. Discussions with pharmaceutical medical liaisons

ALL QUALIFIED RESPONDENTS (S100r1)
Q309 Are you (or the organization where you primarily work) associated with an obesity specialist/weight management practice or center?

1. Yes
2. No
3. Not sure

ALL QUALIFIED RESPONDENTS (S100r1)
Q310 What clinical treatment guidelines (if any) are you aware of for treatment and management of **obesity**?

[MANDATORY TEXT BOX]

ALL QUALIFIED RESPONDENTS (S100r1)
Q310A What clinical treatment guidelines (if any) do you follow for treatment and management of **obesity**?

[MANDATORY TEXT BOX]

ALL QUALIFIED RESPONDENTS (S100r1)
Q311 Which of the following clinical treatment guidelines (if any) do you follow for treatment and management of **obesity**?

Please select all that apply.

[MULTISELECT. RANDOMIZE]

1. Endocrine Society
2. American College of Endocrinologists (ACE) / American Association of Clinical Endocrinology (AACE)
3. American Heart Association (AHA)
4. American College of Cardiology (ACC)
5. American College of Obstetricians and Gynecologists (ACOG)
6. Obesity Medicine Association (OMA)
7. European Association for the Study of Obesity (EASO)
96. Other, please specify [MANDATORY TEXT BOX. ANCHOR]
97. I do not follow any obesity guidelines [EXCLUSIVE][ANCHOR]

[IF FOLLOWS GUIDELINES (Q311r1-96 ANY) ASK Q313. ELSE SKIP TO Q316]

FOLLOWS GUIDELINES (Q311r1-96)
Q313 How effective do you think current clinical guidelines are for treating and managing obesity?

Use a scale where a "1" indicates "Not at all effective," and a "7" indicates "Extremely effective."

Not at all effective					Extremely effective	
1	2	3	4	5	6	7

1. [PIPE IN OPTIONS SELECTED AT Q311]

ALL QUALIFIED RESPONDENTS (S100r1)
Q316 For you, what are the **top 3 biggest barriers** to treating and managing **obesity** in your patients with PCOS?

Please click or drag and drop to rank, starting with '1' meaning "most important," '2' meaning "second most important," and so on until your top three are ranked.

[RANKING QUESTION; RANK TOP 3]

1. Lack of time during patient visit
2. Lack of patient motivation and compliance
3. Patient comorbidities (other than PCOS or obesity)
4. Lack of understanding of link between PCOS and obesity
5. Lack of education on obesity/weight management for providers like me
6. Lack of personal comfort in having weight management discussions with my patients
7. Lack of appropriate treatments (e.g., Rx medications, diet plans)
8. Lack of in-network specialists (e.g., nutritionists, obesity specialists, bariatric surgeons) for referral
96. Other barrier not specified here

RANKED OTHER (Q316r96 IS RANKED IN TOP 3)
Q318 For you, what **other barrier(s)** impact treating and managing **obesity** in your patients with PCOS?

[MANDATORY TEXT BOX]

ALL QUALIFIED RESPONDENTS (S100r1)
Q323 Approximately how often do you discuss weight or weight management with your patients who have PCOS and obesity?

1. Never
2. Sometimes (every few visits)
3. Often (almost every visit)
4. At every visit

[IF DISCUSSES WEIGHT WITH PWPCO (Q323r2-4) ASK Q326. ELSE SKIP TO Q331

DISCUSSES WEIGHT WITH PWPCO (Q323r2-4)
Q326 Which of the following topics do you typically discuss when talking about weight or weight management with your patients who have PCOS and obesity?

Please select all that apply.

[MULTIPLE SELECT. RANDOMIZE]
1. Explain the effect their weight has on their overall health
2. Explain the effect their weight has on their PCOS
3. Explain the effect that their PCOS has on their weight
4. Help them set goals to improve their weight
5. Help them understand why they have excess weight
6. Make them aware of prescription medications that will help them lose weight
96. Other, please specify [MANDATORY TEXT BOX. ANCHOR]

DISCUSSES WEIGHT WITH PWPCO (Q323r2-4)
Q330 For approximately what proportion of your patients with PCOS and obesity do you…?

Your best estimate will do. Your responses do not need to add to 100%.

[RANGE 0-100]

	Proportion of patients with PCOS and obesity
1. Prescribe weight loss medications	|_|_|_|%
2. Refer to a medical weight loss program	|_|_|_|%
3. Suggest general lifestyle modifications (diet and exercise)	|_|_|_|%
4. Suggest a specific type of diet	|_|_|_|%
5. Suggest a commercial weight loss program	|_|_|_|%
6. Suggest referral to weight loss clinic	|_|_|_|%
7. Suggest referral to registered dietitian/nutritionist	|_|_|_|%

ALL QUALIFIED RESPONDENTS (S100r1)
Q331 Please indicate how much you agree with the following regarding prescription medications for weight loss…

Use a scale where 1 means "Completely disagree" and 7 means "Completely agree."

[RANDOMIZE, CAROUSEL]

1 – Completely disagree 2 3 4 5 6 7- Completely agree

1. Prescription weight loss medications are more effective for my patients than other treatment options for weight loss.
2. I am likely to prescribe new prescription weight loss medications in the future.
3. I am more likely to recommend my patients take a prescription medication than have a surgery (bariatric) to lose weight.
4. I am concerned about the side effects associated with prescription weight loss medications.
5. My patients would like me to offer prescription weight loss medication to help them with their weight loss efforts.
6. My patients trust me to recommend a prescription weight loss medication that is right for them.
7. There are good options available today for prescription weight loss medications.
8. Cost is a major barrier for my patients to consider prescription weight loss medications.
9. Patients would rather lose weight on their own than depend on medication.
10. I am likely to review the prescription weight loss medications available with my patients.
11. I am concerned about the long-term safety associated with prescription weight loss medications.
12. I don't know enough about prescription weight loss medications to feel comfortable prescribing them to my patients with obesity.

ALL QUALIFIED RESPONDENTS (S100r1)
Q333 What prescription weight loss medications are you aware of for treating patients with PCOS and obesity?

[LARGE MANDATORY TEXT BOX]

PRESCRIBES ANTI-OBESITY MEDICATIONS (Q330r1>0)
Q335 Which of the following weight loss medications do you currently prescribe to your patients with PCOS and obesity?

Please select all that apply.

[MULTISELECT. RANDOMIZE]

1. Liraglutide (Saxenda)
2. Orlistat (Xenical, Alli)
3. Naltrexone-bupropion (Contrave)
4. Phentermine (Adipex, Suprenza)
5. Phentermine/Topiramate ER (Qsymia)
96. Other, please specify [MANDATORY TEXT BOX. ANCHOR]

PRESCRIBES ANTI-OBESITY MEDICATIONS (Q330r1>0)
Q335A For what reasons do you discontinue pharmacotherapy for weight loss for a patient with PCOS and obesity?

Please select all that apply.

[MULTISELECT. RANDOMIZE]

1. Lack of patient willingness to adhere to therapy
2. Cost to patient / insurance coverage
3. Not seeing desired effects (e.g., % body weight loss)
4. Patient concern over long-term safety
5. Patient exhibiting side effects
6. Patient would like to become pregnant (now or within the next year)
7. I prefer patients use traditional lifestyle modifications

8. Patient achieved desired weight loss goal
96. Other, please specify [MANDATORY TEXT BOX. ANCHOR]
97. I never discontinue pharmacotherapy for weight loss among patients with PCOS and obesity [ANCHOR. EXCLUSIVE]

ALL QUALIFIED RESPONDENTS (S100r1)
Q343 Thinking of your patients with PCOS and obesity, please indicate how much you agree with each of the following…

Use a scale where 1 means "Do not agree at all" and 7 means "Completely agree."

1 - Do not agree at all 2 3 4 5 6 *7 - Completely agree*

[RANDOMIZE, CAROUSEL]

1. My patients could lose weight if they really set their mind to it.
2. I have a responsibility to actively contribute to my patients' weight loss effort.
3. My patients' weight loss is completely their responsibility.
4. For my patients to lose weight, they would need to completely change their lifestyles.
5. My patients are happy with their current weight.
6. My patients are past the point where they can lose weight on their own.
7. My patients are motivated to lose weight.
8. Obesity is less important than many of the other diseases I treat.
9. I do not feel comfortable bringing up a patient's weight unless they mention it first.
10. My patients know how to keep the weight off

ALL QUALIFIED RESPONDENTS (S100r1)
Q346 What are the **top 5 types of support that would be most helpful** <u>for your patients with PCOS and obesity</u> to be successful with managing their weight?

Select your top 5.

[5 SELECTIONS] [RANDOMIZE]

1. Resources for family and friends to help them understand how to be supportive
2. Specific meal plans to follow for weight management
3. Online support groups for those trying to lose weight
4. Local in-person support groups for those trying to lose weight
5. More programs offered at workplaces to help people lose weight
6. Financial support for healthy choices (gym membership, healthy foods)
7. App with weight loss tracking and ideas for healthy eating and physical activity
8. Weekly follow-up visits with a healthcare provider
9. Meetings with dietitian / nutritionist
10. Access to mental health support
11. Access to a physician who specializes in obesity
96. Other, please specify [MANDATORY TEXT BOX. ANCHOR]

ALL QUALIFIED RESPONDENTS (S100r1)
Q350 Please indicate how comfortable you feel addressing **health holistically** (e.g., discussing and treating your patients' PCOS, weight, and comorbidities) among your patients with PCOS and obesity.

Please use a scale from 1 to 7 where "1" means "Not at all comfortable" and "7" means "Extremely comfortable."

Not at all comfortable						Extremely comfortable
1	2	3	4	5	6	7

ALL QUALIFIED RESPONDENTS (S100r1)
Q355A Do you think it is necessary for a woman with PCOS who has excess weight or obesity to lose weight in order for PCOS symptoms (e.g., irregular periods, acne) to improve?

1. Yes
2. No
3. Not sure

THINK WEIGHT LOSS IS NECESSARY FOR SYMPTOM IMPROVEMENT (Q355Ar1)
Q355C What percentage of body weight do you think a woman with PCOS and excess weight or obesity would need to lose in order to improve PCOS symptoms?

|_|_|_| % of body weight necessary to lose

ALL QUALIFIED RESPONDENTS (S100r1)
Q355B Do you think it is necessary for a woman with PCOS who has excess weight or obesity to lose weight in order to improve or restore fertility (e.g., achieve a successful pregnancy)?

1. Yes
2. No
3. Not sure

THINK WEIGHT LOSS IS NECESSARY FOR FERTILITY (Q355Br1)
Q355D What percentage of body weight do you think a woman with PCOS and excess weight or obesity would need to lose in order to improve or restore fertility (e.g., achieve a successful pregnancy)?

|_|_|_| % of body weight necessary to lose

SECTION 400: BENEFITS OF WL AND CLINICAL TRIALS

ALL QUALIFIED RESPONDENTS (S100r1)
Q410 Now, imagine there is hypothetical treatment going into clinical trials to assess improvement in PCOS symptoms among people with obesity. Please indicate how interested you would be in referring your patients with PCOS and obesity to participate in a clinical trial for this hypothetical treatment if you had the opportunity.

Use a scale where a "1" indicates "Not at all interested," and a "7" indicates "Extremely interested."

Not at all interested						Extremely interested
1	2	3	4	5	6	7

ALL QUALIFIED RESPONDENTS (S100r1)
Q415 Still thinking of a hypothetical treatment going into clinical trials to assess improvement in PCOS symptoms among people with obesity, please indicate how **meaningful** each of the following endpoints would be to you in your decision to prescribe the medication.

Use a scale where a "1" indicates "Not at all meaningful," and a "7" indicates "Extremely meaningful."

Not at all meaningful						Extremely meaningful
1	2	3	4	5	6	7

1. Impact of Weight on Quality of Life (IWQOL)
2. Improvement in PCOS symptoms (e.g., regulation of menses, reduced acne, hair growth [Ferriman-Gallwey Hirsutism score])
3. Reduced infertility rate
4. Improvement in associated comorbidities (e.g., decreased A1C, lower blood pressure)
5. Clinical mental health scores (depression/anxiety)
6. 5-10% weight loss
7. 11-25% weight loss
8. Reduction in waist circumference
96. Other, please specify: [MANDATORY TEXT BOX. ANCHOR]

ALL QUALIFIED RESPONDENTS (S100r1)
Q426 What are your expectations on how **obesity management** will be handled for patients with PCOS and obesity in the next 5 years?

[MANDATORY LARGE TEXT BOX]

ALL QUALIFIED RESPONDENTS (S100r1)
Q430 What are your expectations on how **PCOS management** will be handled for patients with PCOS and obesity in the next 5 years?

[MANDATORY LARGE TEXT BOX]

SECTION 100: DEMOGRAPHICS

ALL QUALIFIED RESPONDENTS (S100r1)
Q100 How do you describe yourself?

1. Male
2. Female
3. Transgender
4. Do not identify as female, male or transgender

ALL QUALIFIED RESPONDENTS (S100r1)
Q105 Which description best categorizes the setting where you work most often?

1. Urban
2. Suburban
3. Rural

ALL QUALIFIED RESPONDENTS (S100r1)
Q106 Thinking about where you work most often, what percentage of revenue comes from patients with the following insurance types?

Your total must sum to 100%. Please check the box below if you are not sure.

[RANGE: 0-100; SUM TO 100; CONSTANT SUM; RECODE BLANK TO ZERO]

1. Medicare [_] %
2. Medicaid [_] %
3. Commercial insurance/Private pay [_] %
4. Self-pay/Cash [_] %
5. Other [_] %
6. Not sure [EXCLUSIVE]

ALL QUALIFIED RESPONDENTS (S100/r-4)
Q107 What proportion of your revenue is based on the following types of reimbursement?

Your total must sum to 100%. Please check the box below if you are not sure.

[RANGE: 0-100; SUM TO 100; CONSTANT SUM; RECODE BLANK TO ZERO]

1. Value-based [_] %
2. Fee-for-service [_] %
3. Other [_] %
4. Not sure [EXCLUSIVE]

ALL QUALIFIED RESPONDENTS (S100r1)
Q110 What is your height (feet, inches)?

Your best estimate will do.

[RANGE 1-9]
Feet: |_|
[RANGE 0-11]
Inches: |_|_|

ALL QUALIFIED RESPONDENTS (S100r1)
Q115 What is your current weight (pounds)?

Please be as exact as possible.

[RANGE 50-1000]
Pounds |_|_|_|_|

ALL QUALIFIED RESPONDENTS (S100r1)
Q116 HIDDEN QUESTION FOR BMI CALCULATION

[BMI = (Q115*703) / (Q110r1*12 + Q110r2) ^2]

ALL QUALIFIED RESPONDENTS (S100r1)
Q117 HIDDEN QUESTION FOR WEIGHT CLASSIFICATION

1. Underweight (Q116 < 18.5)
2. Normal Range (Q116 ≥ 18.5 AND Q116 < 25)
3. Overweight (Q116 ≥ 25 AND Q116 < 30)
4. Obese Class I (Q116 ≥ 30 AND Q116 < 35)
5. Obese Class II (Q116 ≥ 35 AND Q116 < 40)
6. Obese Class III (Q116 ≥ 40)
